# Supplementary material for: Cervical transcutaneous vagal neuromodulation in chronic pancreatitis patients with chronic pain: A randomised sham controlled clinical trial
Source: PLoS One. 2021 Feb 26;16(2):e0247653. doi: 10.1371/journal.pone.0247653 (PMC7909707; doi:10.1371/journal.pone.0247653)

**Supporting information S3**

Figure S3: Individual plots of the maximal and average pain scores for the treatment period 1 and 2 for the two sequences (nVNS/sham in black and sham/nVNS in grey).


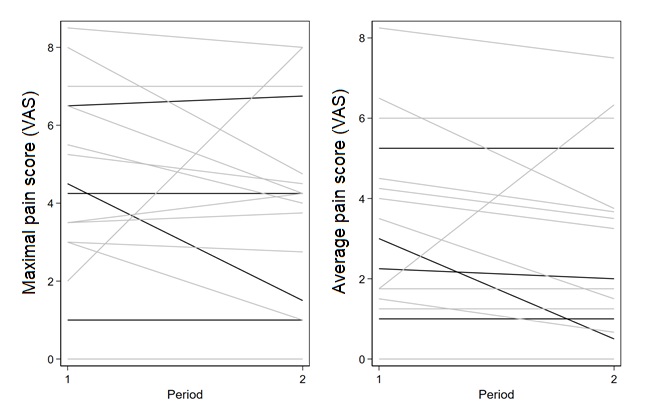

Supplement: S1 Fig — (DOCX) [file pone.0247653.s001.docx]
