# Supplementary material for: Cervical transcutaneous vagal neuromodulation in chronic pancreatitis patients with chronic pain: A randomised sham controlled clinical trial
Source: PLoS One. 2021 Feb 26;16(2):e0247653. doi: 10.1371/journal.pone.0247653 (PMC7909707; doi:10.1371/journal.pone.0247653)
Supplement: S1 Table — (DOCX) [file pone.0247653.s002.docx]

**Supporting information S2**

***Table S2: Demographics by sequences***

| Sequence | nVNS/sham | sham/nVNS |
| --- | --- | --- |
| n (%) | 4 (25) | 12 (75) |
| Age (years) | 55.0±6.4 | 57.2±10.4 |
| Male, n (%) | 4 (100) | 10 (83) |

*Note: Values are means ± SD or percentage. Abbreviations: n=number of patients.*
