# Supplementary material for: Cervical transcutaneous vagal neuromodulation in chronic pancreatitis patients with chronic pain: A randomised sham controlled clinical trial
Source: PLoS One. 2021 Feb 26;16(2):e0247653. doi: 10.1371/journal.pone.0247653 (PMC7909707; doi:10.1371/journal.pone.0247653)
Supplement: S2 Table — (DOCX) [file pone.0247653.s003.docx]

**Supporting information S4**

***Table S4: The primary and secondary clinical endpoints with correction for the baseline assessments.***

|  |  |  |  | |  | |  | |
| --- | --- | --- | --- | --- | --- | --- | --- | --- |
| **Primary clinical endpoints** | **Pain diary** | | | | | | | |
|  | **Variables** | **Sequence** | **Treatment period**  **Mean±SD** | | **Within-individual differences for**  **nVNS vs. sham** | **Treatment effect for nVNS vs. sham (95% CI)** | **P**  **(effect)** | **P**  **(carry-over)** |
|  |  |  | **1** | **2** |  |  |  |  |
|  | Average pain score (VAS) | nVNS/sham | -0.5±0.8 | -0.6±0.5 | 0.06±0.63 | 0.14  (-1.13:1.41) | 0.81 | 0.53 |
|  |  | sham/nVNS | -0.4±1.2 | -0.2±1.5 | 0.08±1.11 |  |  |  |
|  | Maximal pain score (VAS) | nVNS/sham | -1.3±2.7 | -0.6±0.7 | -0.31±1.41 | 0.00  (-1.97:1.97) | 1.00 | 0.72 |
|  |  | sham/nVNS | -1.0±1.8 | -0.4±1.9 | 0.31±1.63 |  |  |  |
| **Secondary clinical**  **endpoints** | **Brief Pain Inventory questionnaire** | | | | | | | |
|  | BPI Pain | nVNS/sham | -0.6±1.8 | -0.3±0.4 | -0.2±0.9 | 0.08  (-1.47:1.63) | 0.91 | 0.45 |
|  |  | sham/nVNS | -0.1±1.3 | 0.4±2.3 | 0.2±1.3 |  |  |  |
|  | BPI Interference | nVNS/sham | -2.0±1.9 | -0.4±0.6 | -0.8±0.8 | -0.83  (-1.99:0.34) | 0.15 | 0.25 |
|  |  | sham/nVNS | -0.2±1.4 | -0.4±1.9 | 0.0±1.0 |  |  |  |
|  | **Quality of life** | | | | | | | |
|  | Global health status | nVNS/sham | 27.1±28.4 | 12.5±8.3 | 7.3±10.4 | 0.69  (-20.3:21.7) | 0.94 | **0.04** |
|  |  | sham/nVNS | 11.8±23.2 | -1.4±17.0 | -6.6±18.3 |  |  |  |
|  | Physical functioning | nVNS/sham | 25.0±25.2 | 0.0±7.7 | 12.5±14.5 | 14.6  (1.3:28.0) | **0.03** | 0.10 |
|  |  | sham/nVNS | 0.1±15.5 | 4.4±11.1 | 2.2±9.5 |  |  |  |
|  | Role functioning | nVNS/sham | 33.3±50.0 | 8.3±9.6 | 11.1±25.5 | 14.6  (-5.7:34.9) | 0.14 | 0.39 |
|  |  | sham/nVNS | 8.3±19.5 | 15.3±20.7 | 3.5±11.5 |  |  |  |
|  | Emotional functioning | nVNS/sham | 18.8±43.2 | 2.1±10.5 | 8.3±22.6 | 5.6  (-12.4:23.5) | 0.52 | 0.49 |
|  |  | sham/nVNS | 7.6±13.0 | 2.1±17.1 | -2.8±11.3 |  |  |  |
|  | Cognitive functioning | nVNS/sham | 16.7±36.0 | 8.3±16.7 | 4.2±10.8 | 3.5  (-11.6:18.6) | 0.63 | 0.33 |
|  |  | sham/nVNS | 4.2±14.4 | 2.8±18.6 | -0.7±12.5 |  |  |  |
|  | Social functioning | nVNS/sham | 8.3±41.9 | 4.2±16.0 | 2.1±15.8 | -2.8  (-16.8:11.2) | 0.68 | 0.81 |
|  |  | sham/nVNS | 8.3±24.1 | -1.4±13.2 | -4.9±9.7 |  |  |  |
|  | Fatigue | nVNS/sham | -22.2±29.4 | -8.3±10.6 | -5.6±9.6 | -4.6  (-26.7:17.5) | 0.66 | 0.53 |
|  |  | sham/nVNS | -11.1±20.1 | -9.3±24.1 | 0.9±16.7 |  |  |  |
|  | Nausea and vomiting | nVNS/sham | 0.0±0.0 | 0.0±0.0 | 0.0±0.0 | 7.6  (-8.7:23.9) | 0.33 | 0.88 |
|  |  | sham/nVNS | -6.9±18.1 | 8.3±16.7 | 7.6±14.8 |  |  |  |
|  | Pain | nVNS/sham | -8.3±16.7 | -8.3±16.7 | 0.0±13.6 | 0.0  (-17.0:17.0) | 1.00 | 1.00 |
|  |  | sham/nVNS | -8.3±21.9 | -8.3±19.5 | 0.0±13.8 |  |  |  |
|  | Dyspnoea | nVNS/sham | -22.2±19.2 | 8.3±16.7 | -16.7±0.0 | -19.4  (-37.3:-1.6) | **0.04** | 0.40 |
|  |  | sham/nVNS | -11.1±16.4 | -16.7±22.5 | -2.8±13.9 |  |  |  |
|  | Insomnia | nVNS/sham | -33.3±33.3 | -8.3±16.7 | -11.1±9.6 | -16.7  (-47.8:14.5) | 0.27 | 0.23 |
|  |  | sham/nVNS | -2.8±33.2 | -13.9±22.3 | -5.6±23.9 |  |  |  |
|  | Appetite loss | nVNS/sham | 0.0±33.3 | -8.3±16.7 | 5.6±9.6 | 13.9  (-13.2:41.0) | 0.29 | 0.43 |
|  |  | sham/nVNS | -25.0±28.9 | -8.3±28.9 | 8.3±20.7 |  |  |  |
|  | Constipation | nVNS/sham | -8.3±16.7 | 0.0±0.0 | -4.2±8.3 | -4.2  (-18.5:10.2) | 0.54 | 0.63 |
|  |  | sham/nVNS | 0.0±24.6 | 0.0±14.2 | 0.0±12.3 |  |  |  |
|  | Diarrhoea | nVNS/sham | 8.3±16.7 | 0.0±27.2 | 4.2±21.0 | 1.4  (-21.0:23.7) | 0.90 | 0.66 |
|  |  | sham/nVNS | 2.8±26.4 | -2.8±22.3 | -2.8±17.2 |  |  |  |
|  | Financial difficulties | nVNS/sham | -33.3±47.1 | 0.0±27.2 | -16.7±23.6 | -18.6  (-39.4:3.2) | 0.09 | 0.15 |
|  |  | sham/nVNS | 0.0±20.1 | -2.8±17.2 | -1.4±15.0 |  |  |  |
| **Secondary experimental**  **endpoints** | **Cardiac-derived parameters** | | | | | | | |
|  | CVT (LVS) | nVNS/sham | 1.0±1.9 | -0.3±1.3 | 0.6±0.6 | 0.74  (-0.24:1.72) | 0.12 | 0.81 |
|  |  | sham/nVNS | 0.0±1.4 | 0.2±2.3 | 0.1±0.8 |  |  |  |
|  | HR (beats/min) | nVNS/sham | -0.9±0.9 | 1.5±5.1 | -1.2±2.1 | -2.28  (-7.56:3.00) | 0.37 | 0.48 |
|  |  | sham/nVNS | -1.2±8.6 | -3.3±7.6 | -1.0±4.7 |  |  |  |

*Note: Comparisons between nVNS and sham treatments with correction for baseline assessments for the two treatment periods shown by treatment sequence (nVNS/sham, n=4 and sham/nVNS, n=12). The difference in pain intensities between the two treatment sequences were calculated for each patient and compared using a t-test for independent samples. One patient had missing data for role functioning, fatigue, dyspnoea, insomnia and appetite loss and one patient had missing data for cardiac vagal tone.*

*Abbreviations: VAS=visual analogue scale. SD=standard deviation. CI=confidence interval. nVNS=non-invasive vagal nerve stimulation. LVS=linear vagal scale. N=number of patients.*
