# Supplementary material for: Cervical transcutaneous vagal neuromodulation in chronic pancreatitis patients with chronic pain: A randomised sham controlled clinical trial
Source: PLoS One. 2021 Feb 26;16(2):e0247653. doi: 10.1371/journal.pone.0247653 (PMC7909707; doi:10.1371/journal.pone.0247653)

CONSORT 2010 checklist of information to include when reporting a randomised trial*

Section/Topic	Item No	Checklist item	Reported on page No
**Title and abstract**			
	1a	Identification as a randomised trial in the title	1
	1b	Structured summary of trial design, methods, results, and conclusions (for specific guidance see CONSORT for abstracts)	3
**Introduction**			
Background and objectives	2a	Scientific background and explanation of rationale	4
	2b	Specific objectives or hypotheses	4,5
**Methods**			
Trial design	3a	Description of trial design (such as parallel, factorial) including allocation ratio	6
	3b	Important changes to methods after trial commencement (such as eligibility criteria), with reasons	NA
Participants	4a	Eligibility criteria for participants	6,7
	4b	Settings and locations where the data were collected	6
Interventions	5	The interventions for each group with sufficient details to allow replication, including how and when they were actually administered	5,6
Outcomes	6a	Completely defined pre-specified primary and secondary outcome measures, including how and when they were assessed	7,8
	6b	Any changes to trial outcomes after the trial commenced, with reasons	NA
Sample size	7a	How sample size was determined	9,10
	7b	When applicable, explanation of any interim analyses and stopping guidelines	9,10
Randomisation:			
Sequence generation	8a	Method used to generate the random allocation sequence	7
	8b	Type of randomisation; details of any restriction (such as blocking and block size)	7
Allocation concealment mechanism	9	Mechanism used to implement the random allocation sequence (such as sequentially numbered containers), describing any steps taken to conceal the sequence until interventions were assigned	7
Implementation	10	Who generated the random allocation sequence, who enrolled participants, and who assigned participants to interventions	7
Blinding	11a	If done, who was blinded after assignment to interventions (for example, participants, care providers, those	7

		assessing outcomes) and how	
	11b	If relevant, description of the similarity of interventions	7,8
Statistical methods	12a	Statistical methods used to compare groups for primary and secondary outcomes	9,10
	12b	Methods for additional analyses, such as subgroup analyses and adjusted analyses	9,10
**Results**			
Participant flow (a diagram is strongly recommended)	13a	For each group, the numbers of participants who were randomly assigned, received intended treatment, and were analysed for the primary outcome	11 & Figure 1
	13b	For each group, losses and exclusions after randomisation, together with reasons	11 & Figure 1
Recruitment	14a	Dates defining the periods of recruitment and follow-up	6
	14b	Why the trial ended or was stopped	NA
Baseline data	15	A table showing baseline demographic and clinical characteristics for each group	22 (Table 1)
Numbers analysed	16	For each group, number of participants (denominator) included in each analysis and whether the analysis was by original assigned groups	22,23, 25 (Table 1,2 and 3)
Outcomes and estimation	17a	For each primary and secondary outcome, results for each group, and the estimated effect size and its precision (such as 95% confidence interval)	11,12, 23 (Table 2)
	17b	For binary outcomes, presentation of both absolute and relative effect sizes is recommended	22 (Table 1)
Ancillary analyses	18	Results of any other analyses performed, including subgroup analyses and adjusted analyses, distinguishing pre-specified from exploratory	25 (Table 3)
Harms	19	All important harms or unintended effects in each group (for specific guidance see CONSORT for harms)	13
**Discussion**			
Limitations	20	Trial limitations, addressing sources of potential bias, imprecision, and, if relevant, multiplicity of analyses	16,17
Generalisability	21	Generalisability (external validity, applicability) of the trial findings	15,16
Interpretation	22	Interpretation consistent with results, balancing benefits and harms, and considering other relevant evidence	14-17
**Other information**			
Registration	23	Registration number and name of trial registry	1,3
Protocol	24	Where the full trial protocol can be accessed, if available	6
Funding	25	Sources of funding and other support (such as supply of drugs), role of funders	19

*We strongly recommend reading this statement in conjunction with the CONSORT 2010 Explanation and Elaboration for important clarifications on all the items. If relevant, we also recommend reading CONSORT extensions for cluster randomised trials, non-inferiority and equivalence trials, non-pharmacological treatments, herbal interventions, and pragmatic trials. Additional extensions are forthcoming: for those and for up to date references relevant to this checklist, see www.consort-statement.org.

Neuromodulation in Patients with Painful Chronic Pancreatitis

A randomized, double blind, sham-controlled, prospective, cross-over, controlled study in chronic pain investigating if a novel vagal neuromodulation approach provides analgesic benefit through central mechanisms in patients with chronic pancreatitis

**Trial Registration**	This trial will be registered at the Danish Health and Medicines Authority and the North Denmark Region Committee on Health Research Ethics.
**Funding:**	The study will be conducted as an investigator-initiated study

Aalborg University Hospital

SYNOPSIS

Protocol identification.: Neuromodulation in CP patients

Medical device: GammaCore; ElectroCore LLC; Basking Ridge, NJ, USA

Expected start of the study: November 2017

Expected termination of the study: June 2018

Research Summary

This study is a randomized, double blind, sham-controlled, cross-over, controlled investigation. The overall objective of the study is to conduct a study of vagal tone and the sensory system (brain activity, sensory testing, and questionnaires) assessing the effect of two weeks' transcutaneous vagal neuromodulation in chronic pancreatitis patients not responding adequately to traditional pharmacological pain treatment, in comparison to the effect of two weeks' sham treatment. The active treatment will be performed using a commercially available and validated device called GammaCore (the active treatment) while the sham treatment will be performed using a sham-device. GammaCore device is a non-invasive neurostimulator that has been approved for the treatment of anxiety, primary headache, including migraine.

The study will begin with a one-week baseline registration period, in which the patients will receive no treatment. Next, the baseline period will be followed by a 2-week treatment period where the subjects will be randomized to either active treatment or the sham treatment. Afterwards, a wash-out period of one week and a second baseline registration period of one week will occur. Finally, in the second treatment period the patients will switch in treatment assignment, meaning that patient who received active treatment in the beginning, will now receive sham treatment and vice versa. During both treatments, the patients will be asked to self-administer one stimulation dose bilaterally to the cervical vagal neck area, three times per day (morning 8 am., afternoon 2 pm, and evening 8 pm), see Appendix, Figure 1.

During the whole study, subjects will be asked to complete a pain diary and several questionnaires. Moreover, at the beginning and end of each treatment period (four times), all subjects will undergo testing which will include magnetic resonance imaging (MRI), quantitative sensory testing (QST), cardiac vagal tone (CVT) and collecting blood samples.

The primary efficacy parameters to be evaluated are clinical pain relief and brain alterations using MRI.

PROTOCOL SIGNATURE PAGE

Investigator's statement: I have read and understood the foregoing protocol and agree to conduct the study in compliance with Good Clinical Practice (CPMP/ICH/135/95), designated Standard Operating Procedures, the Danish Health and Medicines Authority, the Research Ethics Committee in Denmark, and within the principles of the World Medical Association, Declaration of Helsinki amended by the 52nd General Assembly, Edinburgh, Scotland, October 2000, clarified by the General Assembly in Washington 2002, Tokyo 2004, and Seoul 2008 and Fortaleza 2013 as outlined herein.

Jens Brøndum Frøkjær	Professor, MD, PhD,
**Principal investigator & Sponsor**	**Principal investigator's title**
13th September 2017	
**Date**	**Principal investigator's signature**

TABLE OF CONTENT

1 ROLES AND RESPONSIBILITIES 7
TIME SCHEDULE 9
2 BACKGROUND 10
2.2. Increased vagal tone and analgesic effect 11
3 MEDICAL DEVICE 12
3.1 Transcutaneous vagus nerve stimulation (tVNS) – GammaCore® 12
3.1.2 Previous studies on transcutaneous stimulation of cervical branch of the vagus nerve 12
3.1.3 Mode of Action of tVNS 13
3.2 Equipoise (Benefit and Risk) 14
3.3 Clinical trial regulation 15
4 OBJECTIVES OF TRIAL 16
4.1 Objectives 16
4.2 Hypothesis 18
5. INVESTIGATIONAL TRIAL DESIGN 19
5.1 Study Endpoint 19
5.2 Study design 20
5.3 Investigational medical device and manufacturer 20
5.4 Study duration 20
6. PATIENT SELECTION 21
6.1 Number of patients 21
6.2 Setting 21
6.3 Eligibility 21
6.4 Inclusion 22
6.5 Exclusion Criteria 22
6.6 Fit for randomization 23
Interventions 24
6.6 Treatment 25
6.7 Patient withdrawal 25
6.7.1 Side effects, risks, complications, and drawbacks 26
6.7.2 Unacceptable adverse effects 27
6.7.3 Discontinuation criteria 27
7. TREATMENT PROCEDURE 28
7.1 Pretreatment registration 28

7.2 Study visit 1, 2, 3 and 4 ... 28
7.2.1 Magnetic Resonance Imaging (MRI) ... 28
7.2.4 Measure of cardiac vagal tone and blood pressure ... 28
7.2.2 Quality sensory testing (QST) ... 29
7.2.3. Visual Analogue Scales and Pain diary ... 29
7.2.4 Blood for hematology and clinical chemistry ... 30
7.3 Storage of blood samples ... 30
7.4 Study procedure ... 30
7.5 Follow up ... 32
7.6 Study end ... 32
8. ADVERSE EVENT REPORTING ... 33
8.1 Definitions ... 33
8.1.1 Adverse event (AE) and adverse device effect (ADE) ... 33
8.1.2 Serious adverse event and serious adverse device effect (SADE) ... 33
8.1.3 Reporting of serious adverse events / serious adverse device effect ... 34
8.2. Recording of adverse events ... 35
8.2.1. Assessments of adverse event ... 35
8.3 Reports at the end of the study ... 36
9. STATISTICAL METHODS AND DATA ANALYSIS ... 37
9.1 Power calculation and level of significance ... 37
9.2. Justification of sample size ... 37
9.3. Level of significance ... 37
9.4 Method analysis ... 37
9.5 Criteria for the termination of the trial ... 38
9.6 Missing data ... 38
9.7 Deviation from the original statistical plan. ... 38
9.8 The selections of subjects to be included. ... 38
9.9 STATA & SPSS ... 38
10. DATA MANAGEMENT ... 39
10.1 Case Report Forms ... 39
10.2 Source data identification and protection ... 39
10.3 Trial master file ... 39
11. ADMINISTRATIVE PROCEDURES ... 40
11.1 Patient insurance ... 40

11.2 Ethical conduct of the trial 40
11.3 Ethical considerations 40
11.4 Patient information and consent 41
11.5 Study Initiation 41
11.6 Study end report 42
11.7 Trial monitoring 42
11.8 Trial audits and inspections 42
11.10 Finances 42
12. DISSEMINATION 44
13. REFERENCES 45
14. APPENDIX 49

1 ROLES AND RESPONSIBILITIES

SPONSOR AND PRINCIPAL INVESTIGATOR
Jens Brøndum Frøkjær, MD, Professor, PhD
Aalborg University Hospital
Mech-Sense, Department of Radiology
Postobx 365
9100 Aalborg, Denmark
jebf@rn.dk

SUB INVESTIGATOR
Asbjørn Mohr Drewes, MD, Professor, DMSc, PhD
Aalborg University Hospital
Mech-Sense, Department of Medical Gastroenterology
Mølleparkvej 4
DK-9000 Aalborg, Denmark
Telephone No.: (+45) 97663562
Email address: amd@mech-sense.com

SUB INVESTIGATOR
Søren Schou Olesen, MD, PhD
Aalborg University Hospital
Mech-Sense, Department of Medical Gastroenterology
Mølleparkvej 4
DK-9000 Aalborg, Denmark
Telephone No.: (+45) 97663524
sso@mech-sense.com

SUB INVESTIGATOR
Louise Kuhlmann Frandsen, MD, PhD student
Aalborg University Hospital
Mech-Sense, Department of Medical Gastroenterology
Mølleparkvej 4
DK-9000 Aalborg, Denmark
louise.frandsen@rn.dk

MRI RESEARCH ENGINEER
Tine Maria Hansen, MSc(Eng), PhD, postdoc
Aalborg University Hospital
Mech-Sense, Department of Radiology
Postobx 365
9100 Aalborg, Denmark
tine.hansen@rn.dk

VAGAL NEUROMODULATION EXPERTISE
Christina Brock, DVM, PhD, associate professor
Aalborg University Hospital
Mech-Sense, Department of Medical Gastroenterology
Mølleparkvej 4, DK-9000 Aalborg, Denmark
Email: christina.brock@rn.dk

PHD STUDENT
Janusiya Muthulingam, MSc. PhD student
Aalborg University Hospital
Mech-Sense, Department of Radiology
Postobx 365
9100 Aalborg, Denmark
j.muthulingam@rn.dk

TRIAL MONITORING
The Good Clinical Practice Unit at Aalborg and Aarhus University Hospitals
Olof Palmes Allé 15
DK-8200 Aarhus, Denmark
Telephone No.: (+45) 7841 3951

DELEGATED
Isabelle Larsen and Annie Baunwall, ***Research Nurses***
Aalborg University Hospital
Mech-Sense, Department of Medical Gastroenterology
Mølleparkvej 4
DK-9000 Aalborg, Denmark
Telephone No.: (+45) 9932 6245/ 9932 6244
Email address: il@mech-sense.com

Carsten Wiberg Simonsen and Kenneth Krogh Jensen, ***Research Radiographers***
Aalborg University Hospital
Mech-Sense, Department of Radiology
Postobx 365
9100 Aalborg
Emails: cws@rn.dk / kekj@rn.dk

MEDICAL DEVICE SUPPLY
ElectroCore LLC
150 Allen Road, Suite 201
Basking Ridge, New Jersey 07920
USA
Telephone: +1 973 355 6691

LABORATORY

Screening sessions, physical examinations and experimental pain testing will be conducted in the Mech-Sense research laboratory at Aalborg University Hospital. Magnetic resonance imaging (MRI) will be conducted in the department of Radiology at Aalborg University Hospital.

TIME SCHEDULE

The study is expected to be initiated in November 2017 and to be concluded by Summer 2019. A detailed overview is given from the diagram below.

	**Summer - Autumn 2017**	**Winter 2017 - 2018**	**Spring - Autumn – 2018**	**Spring 2019**	**Summer 2019**
**Submitting protocol**					
**Pilots**					
**Recruiting subjects**					
**Conducting study**					
**Data analysis**					
**Writing report**					

2 BACKGROUND

Chronic pancreatitis (CP) remains a major source of morbidity in Northern Europe, with an annual incidence of approximately 10 per 100,000 inhabitants. (Andersen, Pedersen, Scheel, & Worning, 1982) The typical cause is long-term disproportionate use of alcohol, while genetic and hereditary as well as environmental and autoimmune factors also can cause pancreatitis. It is a disease characterized by progressive destruction of the pancreatic gland and is typically characterized by severe abdominal pain (Drewes et al., 2008). As the disease evolves, significant impairment of exocrine as well as endocrine functions also becomes evident. Pain is the most common trait in the symptomatology of CP and up to 90 % of patients with CP have abdominal pain with or without exacerbations (Steer, Waxman, & Freedman, 1995). Within 5 years of diagnosis endocrine and exocrine insufficiencies develop in approximately 50 % and 80 % of patients with CP, respectively. (Andren-Sandberg, Ansorge, Eiriksson, Glomsaker, & Maleckas, 2005; Lieb 2nd, Forsmark, Lieb, & Forsmark, 2009) These conditions are usually managed sufficiently with anti-diabetic treatment and pancreatic enzymes to optimize metabolic and nutritional status, whereas the treatment of pain in CP is more cumbersome and intricate. Thus, the pain in CP is a major burden in terms of psychosocial, physical disability and quality of life.

Analgesic medication is part of the initial pain treatment and often includes opioids in the absence of pathology suitable for endoscopic or surgical interventions. However, opioid-based analgesia often only shows limited effectiveness in these patients and is frequently accompanied by undesirable side-effects. Hence, new treatments to control the pain associated with CP are highly warranted (van Esch, Wilder-Smith, Jansen, van Goor, & Drenth, 2006).

One such option is a treatment called vagus nerve stimulation (VNS), in which short bursts of electrical energy are directed onto the cervical vagus nerve follow by neural activation in the brain. The vagus nerve is a cranial nerve that originates in the brainstem, travels through the neck, and then continues down through the thorax and abdomen. The nerve acts as both a sensory and motor nerve controlling the internal organs, and is also involved in regulation of several brain areas involved in pain and emotional processing (Groves & Brown, 2005). Furthermore, previous studies have shown that vagal nerve stimulation possess analgesic effect in humans and animals.

<u>*Pain mechanisms*</u> in CP are multifactorial in origin (Poulsen, Olesen, Malver, Frokjaer, & Drewes, 2013). The traditional understanding of the mechanisms leading to pain has focused on ongoing inflammation, ductal obstruction and tissue hypertension. Nevertheless, *central sensitization of the pain system* is well documented in CP and it is increasingly accepted to play a prominent role in generation and chronification of pain (Drewes et al., 2008; Woolf & Mannion, 1999). By the use of electroencephalography (EEG) and magnetic resonance imaging (MRI) we have demonstrated changes in the central nervous system (CNS) of these patients indicating neuropathic-like central sensitization and neuroplasticity, as also seen in other chronic pain disorders (Frokjaer

et al., 2011; Frøkjær et al., 2012; Søren Schou Olesen, Frøkjær, Lelic, Valeriani, & Drewes, 2011). Likely, recurrent pancreatic inflammation causes irreversible injury to the pancreatic tissue with continued damage of the pancreatic nerves along with peripheral and central sensitization within the pain neuromatrix (Poulsen et al., 2013). Once the disease has advanced and the neural pathophysiological processes are firmly established with sensitization of central pain pathways, the generation of pain often becomes self-perpetuating and independent of the initial nociceptive drive. Consequently, the management of pain becomes difficult and conventional treatment much less effective. This novel and improved understanding of the pain etiology forms the rationale behind this application and advocates a paradigm shift in pain management of CP and chronic pain in general (S. S. Olesen et al., 2011; Pasricha, 2012).

2.2. Increased vagal tone and analgesic effect

Vagal nerve stimulation (VNS), which has traditionally been used in refractory epilepsy and depression, has in recent human and animal studies shown also to have an analgesic effect (Chakravarthy, Chaudhry, Williams, & Christo, 2015). This is mediated through vagal afferent modulation in the nucleus tractus solitaries, raphe magnus, locus ceruleus, amygdala and periaqueductal grey, which are involved in the descending inhibition of pain (Chakravarthy et al., 2015; Frangos, Ellrich, & Komisaruk, 2015). In functional imaging studies, VNS has shown to affect the "pain network" including the thalamus, hypothalamus, parahipocampal gyrus, and cingulate cortex (Kraus et al., 2013; Ring et al., 2000). In healthy subjects, non-invasive transcutaneous electrical VNS (tVNS) reduced sensitivity of mechanically evoked pain and an inhibition of temporal summation of noxious tonic heat (Busch et al., 2013). Similar in chronic pelvic pain patients, tVNS reduced the pain intensity and temporal summation of mechanical pain (Napadow et al., 2012). Evidence of an analgesic effect has also been reported in fibromyalgia and chronic headache(21). In a trigeminal allodynia rat model, VNS was associated with a pain reduction and a decrease in extracellular glutamate indicating brain metabolites to be involved (Oshinsky, Murphy, Hekierski, Cooper, & Simon, 2014).

Based on this rationale, we propose herein to ***investigate the analgesic effect of tVNS in CP patients and explore the induced changes in the central pain system***, which can be detected using advanced neuroimaging techniques. Hence, by depressing central sensitization and associated hyperalgesia, sustained pain relief and reduction in use of analgesics can likely be obtained, see figure 1. The effect of bilateral tVNS by two-week stimulation of the neck area will be assessed in comparison to the effect of two-week sham treatment. The sham-treatment, will be performed using a sham-device. The sham device was identical in appearance, weight, visual and audible feedback, and user application and control but did not deliver electrical stimulations.

3 MEDICAL DEVICE

3.1 Transcutaneous vagus nerve stimulation (tVNS) – GammaCore®

Active transcutaneous VNS (tVNS) will be administered using a handheld device the size of a mobile phone (GammaCore; ElectroCore LLC; Basking Ridge, NJ, USA), which consists of a battery powered portable stimulator with a digital control user interface that controls the stimulation amplitude and two steel contact electrodes. See Figure 4A.

The GammaCore is a class IIa medical device and non-invasive neurostimulator device that has been approved for the clinical treatment of cluster headache, migraine, and medication overuse headache throughout the European Union, South Africa, India, New Zealand, Australia, etc. (ElectroCore, 2017b; NICE, 2016). At the moment, the primary use of GammaCore is to relieve pain and reduce the frequency of attacks for both headaches and migraine. Hence the device is intended for patients with cluster headache or migraine. Additionally, GammaCore is, granted CE marks for use in the following indications: primary headache, bronchoconstriction, epilepsy, depression, anxiety (ElectroCore, 2017a) and gastric motility disorders. More precisely, GammaCore is approved and indicated for use in adults as an adjunctive therapy to reduce the symptoms of gastric motility disorders and irritable bowel syndrome including nausea, vomiting, and abdominal pain (NCT02388269) as specified in the Declaration of Conformity document, see attached file. The mechanisms behind the pain in irritable bowel syndrome also involves sensitization of the visceral sensory system, and are very similar to the pain mechanisms involved in CP. Furthermore, functional gastrointestinal disorders e.g. chronic pancreatitis and irritable bowel syndrome have common clinical manifestations such as abdominal pain relating to visceral neural sensitization (Grover & Drossman, 2010). Hence, we expect that tVNS would have a beneficial effect as well on chronic pancreatits patients experiencing abdominal pain with no major concerns regarding the safety (All, n.d.; Farmer et al., 2014).

The GammaCore device produces a proprietary low-voltage electrical signal comprising a 5-kHz sine wave burst lasting for 1 millisecond (five sine waves, each lasting 200 microseconds), with such bursts repeated once every 40 milliseconds (25 Hz), generating a 24-V peak voltage and 60-mA peak output current; users could adjust the stimulation amplitude. After applying conductive gel to the two stainless steel contact surfaces, subjects administered one 2-minute stimulation of 1) the neck over the vagus nerve, or 2) the upper abdominal quadrants, which is defined as controlled areas (Silberstein, Mechtler, et al., 2016).

3.1.2 Previous studies on transcutaneous stimulation of cervical branch of the vagus nerve

Vagus nerve activity and vagal tone has shown to be a prognostic marker for several diseases such as ischemic cardiovascular disease (Bigger, Fleiss, Rolnitzky, & Steinman, 1993; Coviello et al., 2013; Lanza et al., 1998; Makikallio et al., 2001), autoimmune diseases (Evrengul et al., 2004; Laversuch et al., 1997; Lindgren,

Stewenius, Sjolund, Lilja, & Sundkvist, 1993; Stein, McFarlane, Goldberg, & Ginzler, 1996; Thayer, 2009) and in chronic pain including musculoskeletal pain (Lange et al., 2011), migraine and cluster headache (Gaul et al., 2016; Kinfe et al., 2015). In a case series of 19 patients with cluster headache, complete resolution of pain was achieved in 47% of cluster attacks. Another study with 50 migraine patients revealed pain relief in 38% of attacks, after using vagal neurostimulation. The effect was measured using visual analogue scale scores for pain (Holle-Lee, Gaul 2016). The findings to the respective diseases do not establish causality of vagus activity for disease development but support the notion that modulation of vagus nerve signals may treat diseases with pain as the main symptom.

Surgically implanted vagus nerve stimulation (sVNS) devices have been shown to be effective (now FDA approved) in the treatment of epilepsy (Handforth et al., 1998) and refractory depression (Nierenberg, Alpert, Gardner-Schuster, Seay, & Mischoulon, 2008).

3.1.3 Mode of Action of tVNS

Transcutaneous vagus nerve stimulation therapy delivers electrical signals to the cervical branch of the vagus nerve. This stimulation activates specific fibers in the vagus nerve bundle and thereby inhibit vagal afferent signals to the trigeminal nucleus radialis (TNC) by releasing inhibitory neurotransmitters within the central nervous system. Additionally, recent studies have shown that the stimulation of the vagus nerve inhibit pain by modulating inhibitory neurotransmitter release, leading to decreased TNC glutamate levels (Gaul, Diener et al. 2016).

Central sensitization of the pain system is well documented in CP and it is believed to play a prominent role in its pain pathogenesis. One of the key involvements in central sensitization is glutamate which is released by nociceptor stimulation. Hence, it is believed that stimulation of vagus nerve will stimulate nucleus tractus solitaries (NTS) causing release of inhibitory neurotransmitters (e.g. GABA, serotonin) which will further decrease the level of glutamate, and then block the perception of pain in the cerebral cortex, see figure 1.

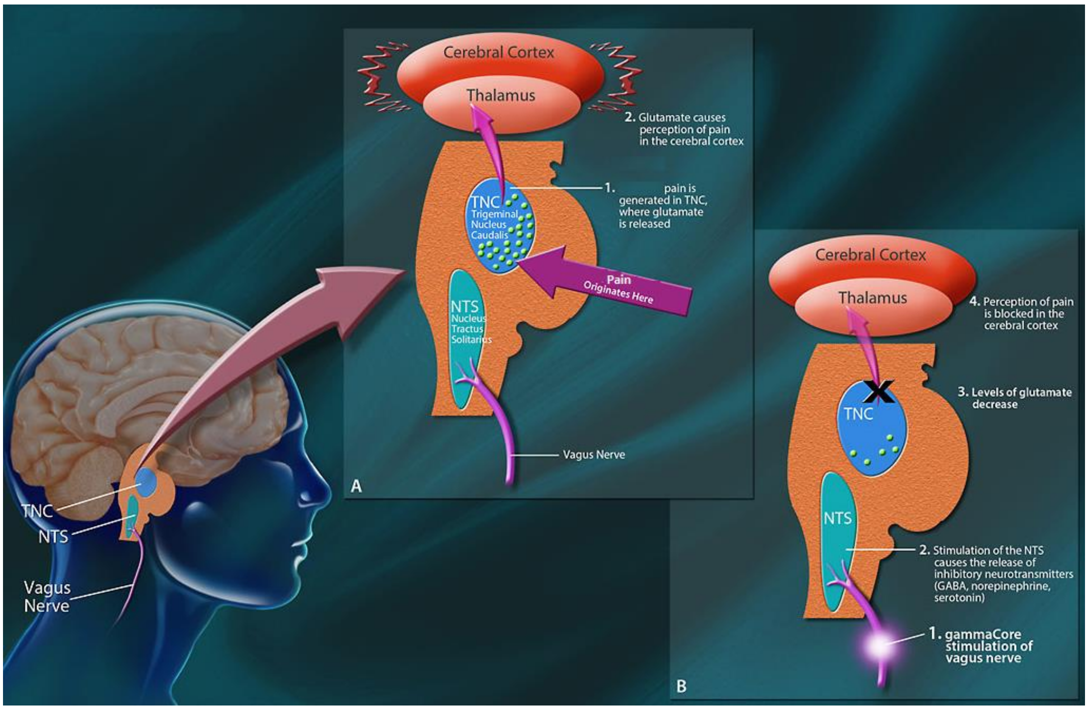

Figure 1 illustrates the mechanisms of tVNS

3.2 Equipoise (Benefit and Risk)

The ethical basis for medical research that involves assigning patients to a novel add-on treatment in this clinical trial has been considered thoroughly. Today pain disorders are challenging to treat without potential severe medical adverse effects and many patients relapse following current medical treatment regimens. The patients can withdraw their consent at any time, and this will not influence their pharmacological treatment.

The GammaCore device is among others CE marked for the treatment of migraine, clusters headache etc. (CE 571753). Historically, stimulation of the vagus nerve was known to cause adverse side effects, including bradycardia and bronchoconstriction. These negative effects were shown to be the result of indiscriminant stimulation of all fibers in the vagal bundle (the vagus nerve is primarily comprised of Aβ and C fibers), which can now be avoided by specifically tuning the electrical signals to selectively stimulate only the Aβ fibers. This is possible because of the difference in electric field strengths necessary to activate the different fiber types. The intensity, pulse duration and frequency of the tVNS stimulation parameters have been optimized to induce signals in the large, myelinated low threshold afferent Aβ fibers of the cervical branch of the vagus, versus the high threshold efferent C-fibers, which innervate the heart, and have potential for adverse cardiac or other systemic, parasympathetic effects. To address the theoretical risk of vagus nerve overstimulation on the heart and airways, a study was conducted at the Lovelace Respiratory Research Institute (Albuquerque, NM, USA) in hypersensitized beagles. Review of heart rate and airway resistance before, during and after

stimulation indicated that there were no significant adverse changes associated with stimulation. These results are consistent with the human clinical experience with the GammaCore device, together with ECG data from over 40 subjects as additional safety data specific to the potential cardiovascular risks of tVNS treatment.

Currently 2000 GammaCore devices have been used in European countries to date. Physicians were asked to voluntarily provide feedback on the outcomes observed in their patients for whom the commercial GammaCore device was prescribed. Patients have safely self-administered stimulation using the GammaCore device between 4 and 20 times per day using a combined prophylactic and acute protocol. In the countries in which the GammaCore is marketed, there have been 167 total complaint reports from sales / distribution (between July 2011 to August 24, 2015) identified through the company's complaint handling system or in discussions with physicians who prescribed the devices for their patients. Seventy-two reports are classified as medical events (of those, 27 are reports of "treatment not effective"). None were device related serious adverse events. Regarding the long-term potential risks, there are up to twenty years of experience with implanted vagus nerve stimulators on which to rely for support. More than 100,000 epilepsy and depression patients have received implanted VNS devices, providing nearly continuous stimulation, and the results have shown neither a heightened risk for significant side effects, nor a loss of efficacy as a result of tachyphylaxis. A recent published study showed significant reductions in 1) patient-perceived pain intensity, 2) mean number of headache days per month and 3) and mean number of migraine attacks per month, in a study where 20 patients were self-treating themselves with the GammaCore in a 3 months study period. No adverse events were observed (Kinfe et al. 2015). Based on these reports, it is our confidence that the side effects or discomfort obtained during stimulation counterbalances the benefits regarding possible analgesic effect. Today these patients receive medical treatments with unwarranted side effects, so this new approach with promising effect on the level on the pain system is a welcomed alternative. Finally, patients are informed that they discontinue the treatment at any point. Furthermore, based on the recommendations from the National institute for Health and Care Excellence (NICE), it appears that the safety of transcutaneous stimulation of the cervical branch of the vagus nerve for the two pain conditions (cluster headache and migraine) raises no major concerns(All, n.d.).

3.3 Clinical trial regulation

The present clinical study will be conducted in compliance with this protocol, the guidelines of the World Medical Association Declaration of Helsinki in its revised edition (Seoul, South Korea, 2008), the guidelines of International Conference on Harmonization (ICH) GCP (CPMP/ICH/135/95), designated Standard Operating Procedures, and with local laws and regulations relevant to the use of new therapeutic agents in the country of conduct.

4 OBJECTIVES OF TRIAL

4.1 Objectives

To investigate whether a novel vagal neuromodulation approach provide an analgesic benefit through central mechanisms in patients with chronic pancreatitis as compared to the sham treatment. Hence, the primary clinical objective is to understand the effect of two-week transcutaneous vagal neuromodulation on the daily pain experienced by patients with CP as documented in a pain diary. Additional clinical objectives are to understand the effect of transcutaneous vagal neuromodulation on quality of life and physical functioning. The primary experimental objective is to assess the effect of vagal neuromodulation on resting state brain function assessed with brain MRI, while the additional secondary objectives are assessment of the sensory system (QST), vagal tone and blood samples. Finally, the relationship between brain (MRI) and sensory (QST) changes in CP patients related to pain will be determined.

Specific aims:

To assess the effect of two weeks' transcutaneous vagal neuromodulation, in comparison to the sham treatment, in CP patients not responding adequately to traditional pharmacological pain treatment on:

Clinical aims:

- **Primary clinical aim**: The perceived clinical pain measured by pain diary.
- **Secondary clinical aim**: Differences in the level of questionnaires regarding pain and quality of life etc.

Experimental aims:

- **Primary experimental aim**: The brain mechanisms involved in chronic pain and central sensitization using MRI technique.
- **Secondary experimental aim:** Sensory changes and differences in cytokines from blood samples.

Data will be collected until 21 patients have been included in the study. Expected termination of the study is at 30.06.2019.

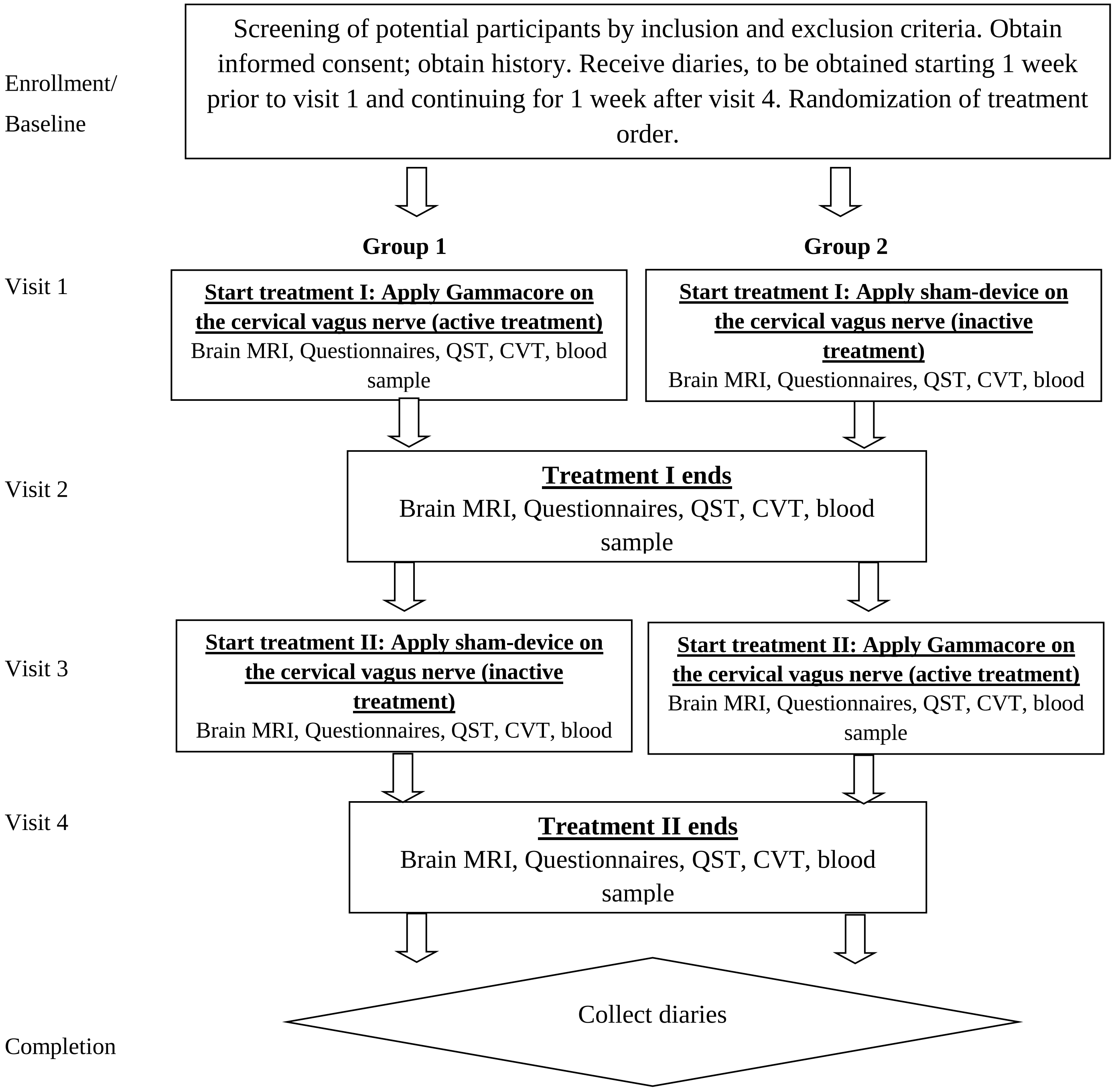

Figure 2: Schematic of the randomized controlled cross-over, controlled study design. QST: Quantitative sensory testing. CVT: cardiac vagal tone. MRI: Magnetic resonance imaging.

4.2 Hypothesis

The hypothesis of the present study is that this neuromodulation approach of the vagus nerve, in comparison to the sham treatment, 1) gives pain relief assessed by a daily pain diary as primary outcome and thresholds to quantitative sensory testing and questionnaires as secondary outcomes, and 2) induces changes in pain associated brain networks assessed by resting-state functional magnetic resonance imaging (RSfMRI) as experimental outcome and in combination with brain metabolite concentrations assessed by magnetic resonance spectroscopy (MRS) and diffusion tensor imaging (DTI) as outcomes.

5. INVESTIGATIONAL TRIAL DESIGN

5.1 Study Endpoint

Primary Clinical Endpoints:

The primary clinical efficacy parameter to be evaluated is pain relief. In the clinical part of the study the efficacy is assessed as changes in the daily experience of pain, which will be measured using a patient pain diary based on the visual analog scale (VAS). Maximum intensity and average daily VAS will be recorded on daily basis.

Secondary Clinical Endpoints:

- Change in quality of life QoLQ.[15]
- Changes in pain and physical functioning composite scores of the modified brief pain inventory-short form (mBPI-sf).[16]
- Patient Global Impression of Change (PGIC).[18]

Experimental Endpoints:

- Primary: Detection of brain changes using magnetic resonance imaging
- Secondary: Quantitative sensory testing (thermal, mechanical and muscle stimulation, including conditioned pain modulation (CPM)), cardiac vagal tone (CVT) and blood samples

5.2 Study design

Our overall goal is to assess the effect of two weeks' transcutaneous vagal neuromodulation in CP patients in a within subject design.

This is a randomized, double-blind, sham-controlled, single-center, cross-over clinical trial of the effect of two weeks' transcutaneous vagal nerve stimulation for pain treatment in CP.

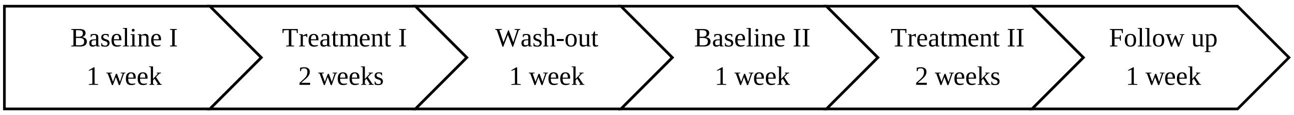

Figure 3 - Schematic flowchart of the interventional study design enabling comparison of the modulatory effect to self-administered transcutaneous vagal nerve stimulation (tVNS) in patients with chronic pancreatitis. All patients will complete 8-weeks pain diary.

5.3 Investigational medical device and manufacturer

Gamma Core

ElectroCore LLC

150 Allen Road, Suite 201

Basking Ridge, New Jersey 07920

USA

Telephone: +1 973 355 6691

The manufacturer does not have any ownership in this project, hence the authors and the manufacturer declare that there is no conflicts of interest regarding the publication of papers in this study.

5.4 Study duration

The study is expected to be initiated in July 2017 and data collection to be concluded

September 2018. A detailed overview is given from the diagram below.

First Patient First Visit (FPFV): November 2017

Last Patient Last Visit (LPLV): June 2019

6. PATIENT SELECTION

6.1 Number of patients

Patients for this study will be enrolled over a period of one and a half year. 21 patients will be included in the study.

6.2 Setting

Screening sessions and physical examinations prior to inclusion will be conducted in Department of Gastroenterology, Mech-Sense, Aalborg University Hospital. The screening will be performed by a medical doctor as well as the inclusion of the patients. All the experiments (MRI, QST, CVT and collecting blood samples etc.) will be conducted at Aalborg University Hospital. Blood samples are stored in a locked research freezer for future analysis at Aalborg University Hospital.

6.3 Eligibility

All eligible patients, who are previously known from the outpatient clinic, will be informed orally and in writing. Additionally, if a potential patient comes to the clinic, the doctor will provide the document "Rekrutteringsbrev" and thereby the doctor will introduce the patient to the study. If the patient is interested to be contacted, he/she has to complete the form from "Rekrutteringsbrev". Afterwards, a physical meeting with a doctor will be scheduled where the patient will get more detailed information about the study. Before the screening meeting, the written information is provided to the patient ("Deltagerinformation"). A medical doctor will conduct the screening meeting according to the Research Ethics Committee's guidelines. The interview will take place in our laboratory at Aalborg University Hospital without any disturbances. During the interview, the patient will be further informed about the project and any questions from the patient will be answered. The patient has the right to consider if he/she is willing to participate in the trial after the information interview (and is encouraged to consider it for a period of approximately 24 hours). The patients are allowed to have an assessor with them to the interview. Before the trial is started the subjects give their informed consent and proxy statement in writing. In the informed consent, the subject can choose not to get any essential information about his/her state of health during the trial. All required standards including "Klinisk afprøvning af medicinsk udstyr til brug på mennesker – God klinisk praksis" will be followed.

All patients will be informed orally and in writing before they decide whether or not they will take part in the study. Furthermore, they will be informed that they are allowed to withdraw from the study at any given time without giving any reason. The patient can contact Janusiya Muthulingam 97 66 51 19 if he/she wants to know the results or any other information about the project.

6.4 Inclusion

Patients will be included after the informed consent and the Power of attorney document "Fuldmagtserklæring" have been signed and a date for first baseline visit will be scheduled.

Inclusion criteria:

- Patients from the ages of 18 with a diagnosis of CP diagnosed using the Mayo Clinic diagnostic criteria.
- The participants must be able to read and understand Danish.
- The patients must suffer from chronic abdominal pain characteristic for CP, meet the criteria for chronic pain (pain ≥ 3 days per week in at least 3 months) and must consider their pain as insufficiently treated with their usual analgesic treatment.
- Personally, signed and dated informed consent document indicating that the patient has been informed of all pertinent aspects of the trial as well as signing the following document: "Informeret samtykke om opbevaring af biologisk material i biobank til fremtidig forskning".
- Personally, signed and dated the Power of attorney document (Fuldmagtserklæring) indicating that the patient has accepted that the Danish Medical Agency (Sundhedsstyrelsen/Lægemiddelstyrelen) have access to the medical records.
- Patients willing and able to comply with the scheduled visits, treatment plan, laboratory tests and other trial procedures.

In order to get all information needed about the patients' disease, clinical information will be passed on from medical records (patient journal) according to Sundhedsloven § 46, stk 1. to the investigator who is responsible for the project. The consent from the patient allows passing on information regarding the participant health, private and other confidential information, so that sponsor and monitor can control the quality of the study in regards to "informationsbekendtgørelsen".

6.5 Exclusion Criteria

- Patients with any clinically significant abnormalities that in the opinion of the investigator may increase the risk associated with trial participation or may interfere with the interpretation of the trial results.
- Alcohol dependence (Alcohol use in accordance with the recommendations by the Danish Health and Medicines Authority are allowed).
- Illegal drug dependencies.
- Participating in another study where investigational drug is used.

- Patients must not suffer from painful conditions other than CP that make them unable to distinguish the pain associated with CP from chronic pain of other origin.
- Cardiovascular diseases
- Low blood pressure < 100/60
- Not able to understand or follow the instructions.
- Any condition with elevated intracranial pressure.
- Female patients who are pregnant or lactating, or intend to become pregnant and male patients who intend to father a child during the course of the study. A pregnancy test will be conducted at baseline and after 8 weeks to ensure that female patients are not pregnant during the study medication period. The investigator will have to urge that fertile female patients use a safe contraception method during the study and for at least 15 hours after termination of the study medication period. The following methods are considered as safe contraception methods:
    - The combined oral contraceptive pill
    - Intra uterine device
    - Gestagen injection
    - Subdermal implantation
    - Hormone vaginal ring
    - Transdermal plaster
- Contraindications for MRI: Such as metallic Foreign Body in the Eye, “Triggerfish” Contact Lens, Gastric Reflux Device, Insulin Pumps, Permanent pacemaker, Temporary external transvenous pacing leads, Other implantable metallic components which is considered unsafe by the medical doctor.
- Previous surgery on vagus nerve.
- Known neuropathy.

6.6 Fit for randomization

When it is ensured that no exclusion criteria are fulfilled, they are included in the study and provided with a randomization ID number, which is kept throughout the entire study. From previous studies, we are highly aware of compliance to the study, but as it is a patient group we will expect approximately 3 of 21 (30%) to drop out. These will be replaced by new patients in order to assure sufficient power. A computer-generated pseudo-random code will be used to assign subjects to order of the treatment. A block randomization will be used, allowing 6 patients at the time to be randomized in equal proportions for the order of active tVNS or active stimulation of the control region.

Interventions

This study consists of two interventions:

1) Transcutaneous vagus nerve stimulation using active GammaCore device (active treatment)
2) Sham treatment using sham-device (inactive treatment)

All patients will participate in both interventions in a randomized order.

After the screening process, obtained informed consent, and randomization process, the patient is successfully included in the study.

While in the hospital setting, patients will be thoroughly instructed in the use of GammaCore, which is accepted as an add-on to existing therapy. Thus, the patient will continue the standard care, without changes in the current treatment. When the healthcare providers are persuaded that the patient is capable to use GammaCore independently, the device will be handed over to the patient.

The patient self-administers the therapy, using GammaCore at home in the morning (8.00 am), afternoon (2.00 pm), and evening (8.pm) for two weeks continually. The patient places the device on the side of the neck, over the cervical branch of the vagus nerve, as carefully instructed by the heath personnel. Hence, the two smooth metal stimulation surfaces are positioned in front of the sternocleidomastoid muscle, over the carotid artery and the stimulation is delivered with the aid of a conductive electrode gel (Sigma gel, Parker Laboratories, NJ, USA), see figure 4A. The patient slowly increases the stimulation strength until small muscle contractions are felt under the skin. Then the patient will receive 120 seconds of sequential transcutaneous stimulation to the left cervical vagal nerve followed by the right cervical vagus nerve to their maximum tolerated amplitude. Hence, both the right site and left site will be stimulated.

The procedures for both interventions are identical, however only the devices are different. For the active treatment, GammaCore device producing a low voltage (peak, 24 V) electrical signal will be used. For the inactive treatment, also called sham treatment, a sham-device producing no electrical signal, will be used. Both devices, will be placed on the side of the neck. An ideal sham-device should mimic the functionality and sensation of active treatment without producing treatment effects or device related adverse events. Hence, to mimic the sensation of the active treatment, the sham-device will provide vibration, when applying on the side of the neck. Since the sham-device provide vibration sensation, we strongly believe that the patients will not identify the active (GammaCore) and inactive (sham-device).

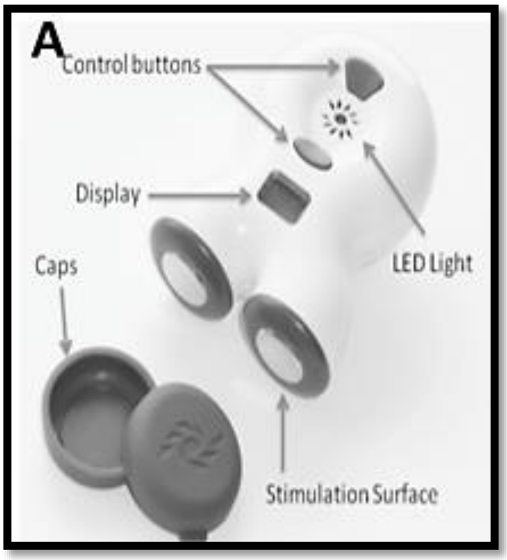

Figure 4A shows the hand held vagal stimulation device, GammaCore

6.6 Treatment

While in the hospital setting, patients will be thoroughly instructed in the use of GammaCore and sham-device, which is accepted as an add-on to existing therapy. As part of the dataset, blood pressure and cardiac vagal tone will be assessed to judge the risk of inducing side effects. The stimulation intensity will be adjusted individually, and this intensity will be used throughout the two-week stimulation. The GammaCore will be handed out for self-administration three times a day (8am, 2pm, and 8 pm). Patients will treat themselves bilaterally (first the right site, then the left site) by applying 2 minutes' stimulation on each side of the neck (or the upper abdominal control region), three times a day for the two weeks.

Even though the procedure is safe, we advise patients to only stimulate themselves when another person (family or friend) are around, due to the low risk of fainting.

6.7 Patient withdrawal

Based on a medical judgment, subjects may be withdrawn at any time.

6.7.1 Side effects, risks, complications, and drawbacks

There is no significant risk associated with the transcutaneous vagal nerve stimulation, however subjects can experience transient symptoms including:

- Shortness of breath and hoarseness/change of voice during stimulation
- Mild pain and muscle twisting during the 2-minute stimulation
- Tingling, pricking on the skin where the device is placed
- Minor skin irritation due to the conductive gel
- Very rare fainting (syncope) is observed

Previous studies (Silberstein, Calhoun, et al., 2016; Yuan & Silberstein, 2017), have used sham-device to test whether transcutaneous vagus nerve stimulation have any effect. So far, no studies have reported any side effects or risks related to the sham device. Since, the sham-device do not produce any electrical stimulation, we strongly believe that it is far safer to use sham-device compared to GammaCore device.

<u>Potential risks – MRI</u>

MRI is a medical imaging procedure that uses strong magnetic fields and radio waves to produce images of internal structures in the body. There are no known harmful side-effects associated with temporary exposure to strong magnetic fields during imaging. However, potential risks of MRI include the following safety concerns: 1) Implanted medical devices that contain metal (e.g. pacemakers, artificial limbs) may malfunction or heat up during imaging, 2) any loose metal object may cause damage or injury if it gets pulled toward the magnet, 3) medication patches can cause a skin burn, 4) dyes from tattoos or tattooed eyeliner can cause skin or eye irritation, and 5) prolonged exposure to radio waves during the scan could lead to slight warming of the body. Further, the MRI equipment is very noisy. Being in an MRI scanner can cause claustrophobia in some people. To minimize risks associated with the MRI exam, we will: 1) ensure that participants do have any counter indications for MRI, such as a pacemaker or other implanted devices; 2) familiarize participants with the noisy and potentially claustrophobia inducing conditions and assure them that they can use an emergency squeeze ball to stop the exam if they feel uncomfortable; 3) provide earplugs to reduce the noise; 4) talk to participants regularly while in the control room via an intercom to check if participants are okay; 5) assure participants that they can stop any time they wish.

Potential Risks – QST

Risks related research protocols for the applied pain stimuli are all previously approved by the Research Ethics Committee (N-2010046, N-20090008). The applied stimuli are all light and incapable of causing tissue damage and will be applied to the skin only. The investigators have worked thoroughly with the experimental stimuli in the past and have never experienced any serious complications inflicted by the stimuli.

QST is used for the noninvasive evaluation of sensory nerve function. These tests can assess and quantify the amount of physical stimuli required for sensory perception involved in pressure, pain, and thermal sensation. Pressure pain thresholds (PPTs) are performed with calibrated pressure algometers (Somedic AB, Stockholm, Sweden). Pressure is applied to the skin and slowly increased until the participant says it is painful, at which point pressure is removed. There is a chance that there will be some residual pain after PPT testing. To minimize risks associated with the QST exam, we will: 1) allow participants to stop the stimulation at any time if she finds it too uncomfortable; 2) not exceed temperatures or pressures that participants describe as intolerable; 3) assure participants that they can stop any time they wish.

Potential Risks – Blood samples

When taking blood samples risk of bruises and infection may occur.

6.7.2 Unacceptable adverse effects

If unacceptable adverse effects are experienced by the device, the patient should contact the investigator. Based on a medical judgment, the study can be discontinued without any consequences for the patient and his/hers ongoing and future treatment.

6.7.3 Discontinuation criteria

A subject must be withdrawn from the trial if the following applies:

1) Withdrawal of informed consent
2) If the participants are not able to tolerate the transcutaneous vagal nerve stimulation.
3) Pregnancy

Otherwise discontinuation will rely on the discretion of each participant. A patient that discontinues will always be asked about the reason(s) for discontinuation and the presence of any adverse events. If needed, they will be seen and assessed by investigator. Withdrawals and dropouts will be replaced by including new patients.

7. TREATMENT PROCEDURE

7.1 Pretreatment registration

- MRI scan
- Cardiac vagal tone
- QST
- Blood samples

7.2 Study visit 1, 2, 3 and 4

CP patients are randomly assigned to one of the two following scenarios, see Figure 2:

1) Transcutaneous vagal nerve stimulation (active treatment) as treatment I, followed by sham treatment (inactive) as treatment II.
2) Sham-treatment (inactive) as treatment I, followed by transcutaneous vagal nerve stimulation (active) as treatment II.

During the whole study, all patients will complete 8-week pain diary, see figure 2 and 3.

Participation in the study will involve four identical study visits (at the beginning and end of both treatment periods). All study visits consist of a 1) brain MRI scan, 2) measuring cardiac vagal tone (CVT), 3) quantitative sensory testing (QST), as well as 4) collecting blood samples. All clinical examinations are performed in the same order for all CP patients. Finally, these patients will complete questionnaires e.g. quality of life (EORTC-QLQ-C30), modified brief pain inventory-short form (mBPI-SF) and Patient Global Impression of Change (PGIC).

7.2.1 Magnetic Resonance Imaging (MRI)

The MRI session will consist of 1) an anatomical 3D scan, 2) a resting state functional MRI scan in which the participant will be instructed to relax with eyes closed. Furthermore, 3) whole brain DTI for assessment of microstructure and fiber tracts, and 4) MR spectroscopy assessment of metabolite concentrations in the thalamus, cingulate cortex, and insula (28) will be performed. The time in the MRI scanner will be about 1hr15min. Heart rate, respiration rate, will be recorded throughout the MRI scan.

7.2.4 Measure of cardiac vagal tone and blood pressure

Subjects will be allowed to rest in the sitting position for 5 minutes after entering the examination room following which three ECG electrodes (Ambu Blue Sensor P, Denmark) will be placed in right and left sub-

clavicular areas and cardiac apex. A non-invasive cardiac monitor will then be attached to the electrodes and the ECG will be sampled at a rate of 8kHz for 5 minutes using a commercially available biosignals acquisition system (Neurozoid, ExtremeBiometrics, London, UK), from which CVT will be derived. Autonomic parameters will be recorded according to internationally agreed recommendations [10]. Blood pressure will be measured non-invasively on the upper arm using a commercially available device (Omron M4, Hoofddorp, Netherlands). These measures, i.e. ECG and blood pressure, are based upon standard routine clinical techniques and it is anticipated that the whole protocol will take around 10 minutes.

7.2.2 Quality sensory testing (QST)

We will perform a short standardized QST session that includes temporal summation, pressure pain thresholds (PPT) and conditioned pain modulation (CPM).

1) **Temporal summation:** Recording of temporal summation to repetitive stimulations in the pancreatic area (T10) and control area (dominant forearm) using the PinPrick stimulator, 256 mN (MRC Systems GmbH Medizintechnische Systeme, Germany).
2) **Pressure pain threshold (PPT):** The pressure pain tolerance threshold (PTT) will be determined by pressing an electronic pressure algometer (Somedic AB, Stockholm, Sweden) on the muscle group. Particularly, C5 - clavicula, TH10 – dorsum, TH10 – abdomen, L1 – spina iliac ant sup, rectus femoris, and bone pressure will be stimulated. The probe has a surface area of 1cm2. Pressure will be increased at a rate of 30 kPa/sec until the PTT is reached.
3) **Conditioned pain modulation (CPM):** Conditioned pain modulation (CPM) is a clinically measurable form of descending pain modulation that can be induced experimentally by a conditioning stimulus (the cold pressor test) and quantified by applying a "test-pain" (pressure stimulation of the right quadriceps musculature) before and after its induction. The difference in pressure stimulus intensity (pain threshold) before and after induction of cold pressor pain for two minutes provides a quantitative index of CPM capacity in the individual patient. The techniques used for pressure stimulation and cold pressor test described above will be combined to measure CPM. In this particular test, the subject will lower the hand in ice water (approximately two degrees Celsius for two minutes).

7.2.3. Visual Analogue Scales and Pain diary

A VAS scale validated for experimental pain will be used for assessments of the experimental endpoints. This scale has previously been shown to be reliable and robust to discriminate experimental sensations. This VAS will be used to assess both non-painful and painful sensations in skin, bone and muscle. It is presented as a 10-cm line, anchored by verbal descriptors: The intensity of the non-painful sensations will be scored with the

following descriptors added to facilitate the scoring: 0 – no pain and 10 – Unbearable pain. This part of the scale is colored red for a clear visual separation of the non-painful and painful range of sensations. The score is measured from the zero-anchor to the patient's mark.

Patients will be asked to score daily pain levels in the diary for 8 weeks, with one VAS value for the average pain over the previous 24 hours and a second VAS value for the worst pain over the previous 24 hours. Use of as need pain medication will also be recorded in the diary.

7.2.4 Blood for hematology and clinical chemistry

2 x 9 ml blood in serum tubes2 x 4 ml EDTA blood, 1x 3 ml citrate-fluoride blood and 1 x 4 ml Li-heparin blood samples are collected and assayed at department of Clinical Biochemistry Aalborg University Hospital, according to laboratory guidelines. Blood collected in serum tubes, will be centrifuged, and serum will be aliquoted and frozen at -80^{0}C. The blood will be saved so that the analyzing can happen at the same time. The following inflammatory state and macrophage markers will be assessed: IFN-G, Il-8, IL-10, IL-12p70, IL-13, IL-1b, Il-2, IL-4, IL-6, IL-8, TNF-α, MCP-1, hs-CRP, sCD163, sMR, H01, neopterin, glucose. Hereafter the samples will be anonymous and saved for five years where after it will be destructed.

7.3 Storage of blood samples

Blood samples are stored in a locked research freezer for future analysis, hence a biobank will be created. All samples will be kept encoded until they are analyzed. Remaining biological material will be anonymous and then stored 5 years for potential future explorative analyses. After the five years, they will be destructed.

7.4 Study procedure

Screening (Visit 0): Screening of potential participants by inclusion and exclusion criteria. Obtain informed consent; obtain history. Instruction to diary will be given. Patients will receive diaries, to be completed starting 1 week prior to visit one and continuing for 1 week after visit 4.

Baseline, day 1-7: Recording of daily pain intensity in the pain diary will begin. Thus, patients will be asked to score daily pain levels in the diary, with one VAS value for the average pain over the previous 24 hours and a second VAS value for the worst pain over the previous 24 hours. Use of as need pain medication will also be recorded in the diary.

Day 8 (Pre-treatment I, visit 1): Ensure that the informed consent is obtained. After this is secured physical examination is conducted, blood samples drawn and patients' medical history will be recorded. The pain

intensity and quality of life are recorded with appropriate questionnaires as described above. Furthermore, MRI, CVT, QST will be carried out during the visit. The above mentioned will take place at the Aalborg University Hospital. Self-administered stimulation (**Treatment I**) in the afternoon and evening will take place in the patients' home.

Examinations for visit 1:

- Ensure that informed consent is obtained.
- Physical examination including weight measurement.
- General and specific medical history (including drug/alcohol/tobacco consumption).
- Questionnaires (QOLQ, mBPI-sf, and PGIC) are filled out.
- Blood for hematology and clinical chemistry.
- Pregnancy test (urine) will be done in females not postmenopausal for more than 1 year or not sterile. Blood pressure measuring.
- GammaCore instructions.
- MRI scan
- CVT
- QST

Day 9 (telephone interview): A study delegated research staff will call the patients to discuss any difficulties in self-administered stimulation. Self-administered stimulation at home in the afternoon and evening. Pain diary will be filled out.

Day 10 – 21: 3 Self-administered bilateral stimulation at home in the morning (8 am), afternoon (2pm), and evening (8pm). Day 21 is the last day of self-administered stimulation. During this period registration of pain diaries will continue.

Day 22 (Post-treatment I, visit 2): The patient will meet at the clinic. MRI scan, QST tests, CVT and blood samples will be collected. Furthermore, the pain and quality of life questionnaires will be filled out. No stimulation. **Wash-out period starting**. During this period registration of pain diaries will continue.

Day 23- 28: **Wash-out period continues.** No stimulation. During this period registration of pain diaries will continue.

Day 29-35: One week **baseline registration**. No stimulation. During this period registration of pain diaries will continue.

Day 36 (Pre-treatment II, visit 3): Meeting at the clinic. MRI data, QST data, CVT data and blood samples will be collected. Self-administered stimulation (**treatment II**) at home in the afternoon and evening.

Contacting the patient through telephone in order to discuss any difficulties in self-administered stimulation. Pain diary will be filled out.

Day 37-49: 3 **Treatment II continues**. Self-administered bilateral stimulation at home in the morning, afternoon and evening. Day 49 is the last day of self-administered stimulation. During this period registration of pain diaries will continue.

Day 50 (Post-treatment II, visit 4): The patient will meet at the clinic. MRI scan, QST tests, CVT and blood samples will be collected. The patients have to complete pain dairy one week further.

Day 50-56: **Follow-up**. Complete pain diary.

Every day the patients will receive an automatic text message (SMS), as a reminder to use the device.

7.5 Follow up

After treatment II, the patient will be followed for one week. Even though the treatments has ended, the patients are still asked to complete the pain diary. Additionally, follow up will be done by telephone interview 1 weeks after treatment.

7.6 Study end

The study ends when last patient has submitted the pain diary, meaning one week after treatment II. Data will be analyzed after reaching a dataset of 21 patients.

8. ADVERSE EVENT REPORTING

The methods for collecting and reporting adverse events are described below.

8.1 Definitions

The definitions of adverse events (AEs), adverse device effect (ADEs), are given below. It is of the most importance that all staff involved in the study is familiar with the content of this section. The principal investigator is responsible for ensuring this. Furthermore, The Principal Investigator or Sponsor and the Manufacturer of GammaCore are responsible for reporting any serious adverse event (SAE) and serious adverse device effect to the Danish Health Authorities, by use of the template "Manufacturer´s Incident Report". The scheme must be sent to the following address: Med-udstyr@dkma.dk

All types of adverse events (incl. AE, ADE, SAE, SAE – near incidents, SADE) will be handled in accordance to "Vejledning til ansøgning om tilladelse til klinisk afprøvning af medicinsk udstyr"

8.1.1 Adverse event (AE) and adverse device effect (ADE)

An adverse event (AE) is the development of an undesirable medical condition or the deterioration of a pre-existing medical condition following or during exposure to a medical device, whether or not considered causally related to the product. An undesirable medical condition can be:

1) Symptoms (e.g., dizziness)
2) Signs (e.g., tachycardia)
3) Abnormal test results of an investigation (e.g., laboratory findings, heart rate variability).

In clinical studies, an AE can include an undesirable medical condition occurring at any time. If any causality between the AE and the investigational transcutaneous vagal nerve stimulation is expected the AE is termed an adverse device effect (ADE). The Summary of Product Characteristics of the GammaCore will be used as reference document to for AEs and ADEs. These are included in the protocol as an appendix.

8.1.2 Serious adverse event and serious adverse device effect (SADE)

A **serious adverse event (SAE)** is an adverse event occurring during any study phase fulfilling one or more of the following criteria:

- Results in death
- Is immediately life-threatening
- Requires in-patient hospitalization or prolongation of existing hospitalization
- Results in persistent or significant disability or incapacity

- Important medical events that may not result in death, be life-threatening, or require hospitalisation may be considered an SAE when, based upon appropriate medical judgement, they may jeopardize the patient and may require medical or surgical intervention to prevent one of the outcomes listed in this definition.

Serious Adverse Events - near incidents: serious adverse events also includes situations in which the incidents mentioned above could have occurred if suitable action had not been taken or if circumstances had been less fortunate.

Serious Adverse Events - malpractice: in addition, a serious adverse event can be an incident caused by imprecise or incomplete results from diagnostic equipment leading to incorrect diagnosis, delayed diagnosis or delayed or incorrect treatment and then the above-mentioned incidents will occur.

Serious adverse device effect (SADE): Adverse device effect that has resulted in any of the consequences characteristic of a serious adverse event. The causality of serious adverse events (i.e. their relationship to study treatment) will be assessed by the investigator(s), who in completing the relevant case report form must answer "yes" or "no" to the question: "Do you consider that there is a reasonable possibility that the event may have been caused by any of the following: – study treatment? – other treatments"?

8.1.3 Reporting of serious adverse events / serious adverse device effect

Investigators and other site personnel must immediately inform sponsor of any SAE / SADE that occurs in the course of the study with the exception of serious incidents that according to the trial protocol or Investigator's Brochure are incidents which do not require to be reported immediately. Reporting must be followed-up by a detailed report in writing, and in both the immediate report and the subsequent report, the investigator must identify the trial subjects with a personal code number. Moreover, the investigator must report to the sponsor any incidents and/or irregular analysis results that according to the trial protocol are critical to the trial subjects' safety. Reporting must be done in compliance with the rules and deadlines stated in the protocol. When reporting deaths, the investigator must submit all the additional information the sponsor may request.

The investigator/sponsor and manufacturer should expedite SAE/SADE´s to the Danish Health Authorities. However, it is the sponsor's responsibility to ensure that all relevant information about SAE and SADE is recorded and reported to the Danish Medicines Agency as soon as possible, and no later than 7 days after the sponsor of such suspected adverse reaction. The reporting to the Danish Medicines Agency will done by fulfilling the "Sundhedstyrelsens indberetingsskema" and by fulfilling " DNVK's indberetningsskema" to the Region Committee on Health Ethics.

No later than 8 days after reporting, must the sponsor inform the Danish Medicines Agency of relevant follow-up information on the sponsor's and the investigator's follow-up action to the reporting. Furthermore, any

other suspected unexpected serious adverse reactions must be reported to the Danish Medicines Agency no later than 15 days from the time when the sponsor is informed about them. All reports will be accompanied by comments and possible consequences for the trial.

The sponsor reports all SAEs / SARs to the Regulatory Authority and The North Denmark Region Committee on Health Ethics once a year together with a security report. Follow-up information on SAEs / SADEs must also be reported by the investigator to the sponsor. If a non-serious AE becomes serious, this and other relevant follow-up information must also be provided to sponsor as described above. All SAEs / SADE s will be recorded in the CRF.

All types of adverse events will be notified to the manufacturer Electrocore and to the Danish Health Authorities by use of Manufactures incident report form.

8.2. Recording of adverse events

Information about adverse events will be collected from the signing of informed consent and in all following contacts with the study-subject through-out the project. Adverse events spontaneously reported by the patient and/or in response to an open question "have you had any health problems during the study since previous visit" from the study personnel or revealed by observation will be recorded at each visit from the first administration of any investigational product until the end of the study.

Any deterioration in laboratory values and vital signs need not be reported as AEs. However, abnormal values that meet criteria for an SAE or cause discontinuation of investigational product must be reported as SAE or AE and recorded in the CRF. The time/date when the AE started and disappeared, maximum intensity, action taken with regard to investigational product, causality rating (yes or no), outcome, if AE caused patient to discontinue the study and whether or not it constitutes an SAE will be reported in the CRF for each AE.

8.2.1. Assessments of adverse event

The causality of non-serious AEs (i.e., their relationship to investigational product) will be assessed in the same way as for SAEs.

The intensity rating is defined as:

1 = mild (awareness of sign or symptom, but easily tolerated)
2 = moderate (discomfort sufficient to cause interference with normal activities)
3 = severe (incapacitating, with inability to perform normal activities)

It is important to distinguish between serious and severe AEs. Severity is a measure of intensity whereas seriousness is defined by the criteria as described above. An AE of severe intensity need not necessarily be considered serious. For example, nausea that persists for several hours may be considered severe nausea, but

not a SAE. Any AE not resolved at the last assessment or discontinuation of the study will be followed up within two weeks and thereafter as long as the investigator consider necessary.

8.3 Reports at the end of the study

The study ends when last patient had the last visit. At the end of the study, sponsor will report all AEs, SAEs and SADEs in a final report for the Regulatory Authority and The North Denmark Region Committee on Health Research Ethics. After the end of the study biological material will be transferred to a research biobank.

9. STATISTICAL METHODS AND DATA ANALYSIS

9.1 Power calculation and level of significance

The power in a clinical trial is the statistical probability that the trial outcome will reject the null hypothesis (there is no difference in the daily experience of pain, measured by pain diary based on the visual analog scale (VAS) between treatment I and treatment II). This is also known as the probability of not committing a Type II error. The power is in general a function of the possible distributions, often determined by a parameter, under the alternative hypothesis. As the power increases, the chances of a Type II error occurring decrease. The probability of a Type II error occurring is referred to as the false negative rate (β). Therefore, power is equal to 1−β, which is also known as the sensitivity. In this exploratory study power, has been set to 80%.

9.2. Justification of sample size

As this is an exploratory study it can be difficult to estimate the sample size. However, based on the previous interventional and neuroimaging studies with resting state fMRI as the primary experimental outcome, 12-17 patients seem to be sufficient to show any clinical and brain changes, see Niesters et al 2012.

The study is powered to detect a minimal difference between the groups of 30% on the average pain diary score during the two weeks of study treatment (primary clinical endpoint). On the basis of a standard deviation of 1.5 we determined that a study with 15 patients are needed in the cross-over design to provide a power of 80%, with the use of a two-sided significance level of 0.05. Hence, in order to minimize the risk of under-powering the study the sample size is set at 21 patients to allow for possible technical obstacles, which can distort the data or possible dropouts.

9.3. Level of significance

The null hypothesis is no significant difference between treatment I and treatment II. The experimental hypothesis is proven significant difference between the treatment I and treatment II, with a 5% significance level.

9.4 Method analysis

General statistical approach: To address differences between the two treatments, we will perform general linear model (GLM) analyses comparing the two groups, with age and gender as covariate.

The principal analysis of clinical endpoints will be by intention-to-treat, meaning that all randomized patients are included in their initially assigned study arm, regardless of adherence to study protocol. Experimental endpoints will be by per-protocol, meaning that only patients completing the experimental setup are included. The primary endpoints will be compared between the treatment groups.

Analysis of MRI data: The PI has experience analyzing all aspects of MRI data (e.g. (Frokjaer et al., 2011, 2013; Frøkjær et al., 2012; Hansen, Olesen, Simonsen, Drewes, & Frokjaer, 2014)). We will use independent component analysis (ICA) with Group ICA of fMRI Toolbox (GIFT; http://icatb.sourceforge.net/groupica.htm) in order to examine resting state network function in addition to the mass-univariate analyses. We will use standard preprocessing procedures in SPM (http://www.fil.ion.ucl.ac.uk/spm/). Moreover, we will use a mixed effects design in which within-subject effects between treatment I and treatment II (before and after both treatments) responses brain activity and group effects will be modeled.

The rest of the data, like demographic data, changes in circulating cytokines, and others, will be used descriptively and as input to regression and mixed model analysis. The final statistical analysis plan, providing details of the analysis and presentation of the results will be finalized before initiating any statistical analysis. Statistical analysis will be performed only after the lock of the database.

9.5 Criteria for the termination of the trial

The trial is terminated when 21 patients with valid data are recorded. If the study fails to recruit 21 patients by June 1st 2019, the study will still be terminated at medio 2019.

9.6 Missing data

Data will be analyzed by the intention-to-treat (ITT) approach providing unbiased comparisons between the patient groups.

9.7 Deviation from the original statistical plan.

It will be reported if there are any deviations from the original statistical plan.

9.8 The selections of subjects to be included.

All randomized subjects will be included if sufficient data exist.

9.9 STATA & SPSS

All statistical analysis will be carried out in STATA and SPSS, which is a general-purpose statistical software. Stata's capabilities include data management, statistical analysis, graphics, simulations, and custom programming.

10. DATA MANAGEMENT

The study will be submitted to the Danish Data Protection Agency through the umbrella application of The North Region Denmark ("Region Nordjyllands´s Paraplyanmeldelse ved Datatilsynet – Sundhedsvidenskabelig forskning i Region Nordjylland". Information about the subjects is protected by the "Lov om behandling af personoplysninger og Sundhedsloven". For each subject a Case Report Form is kept in which data for the subject is entered.

10.1 Case Report Forms

Case report forms (CRF) will be stored at Aalborg University Hospital, Department of Radiology, for 5 years after the end of this study. Digitalized data are backed up on the U specific drive under the responsibility of Jens Brøndum Frøkjær. All forms are filled out during (or immediately after) the assessment of a subject and must be legible. Errors are crossed out, corrections are added and next to the changes date and initials are applied.

10.2 Source data identification and protection

Patient Identification List containing patient number, full name, social security number, study medication and treatment codes for all persons included in the study is created, and ethics according to Komitéloven §20, stk 4 is ensured: *Forsøgspersonens ret til fysisk og mental integritet samt retten til privatlivets fred respekteres og oplysninger vedrørende forsøgspersonen beskyttes efter lov om behandling af personoplysninger.*

The list is populated and updated by a project nurse or other competent person and be stored at

Aalborg University Hospital, Department of Radiology, under the responsibility of the investigator. Principal investigator must maintain complete and accurate records to ensure that the execution of the study is fully documented and the study data can be subsequently verified. These documents should be classified in 2 separate categories: (1) researcher Trial Master File and (2) study/subject's clinical source documents (CRF).

10.3 Trial master file

The trial master file must contain the protocol/amendments, correspondence with the Danish Health and Medicines Authority and The North Denmark Region Committee on Health Research Ethics, informed consent, staff curriculum vitae, forms and other appropriate documents/correspondence etc. Principal investigator Jens Brøndum Frøkjær allows direct access to all source data and documents at monitoring, auditing and inspection from the North Denmark Region Committee on Health Research Ethics, the Danish Health and Medicines Authority or from other countries' health authorities.

11. ADMINISTRATIVE PROCEDURES

11.1 Patient insurance

Clinical responsibility befalls Aalborg University Hospital, and therefore, all subjects will be covered by the patient insurance of Aalborg University Hospital. Participation in a trial involving medical treatment may alter private insurance status. Participants are advised to seek consultancy from their insurance agency if they plan to travel during participation in the study.

11.2 Ethical conduct of the trial

The trial will be performed in accordance with the protocol, International Conference on Harmonization Good Clinical Practice guidelines (IHC GCP), and applicable local regulatory requirements and laws. The trial has been submitted for the Central Denmark Region Committee on Health Research Ethics.

11.3 Ethical considerations

The participants may not obtain any direct personal benefit. On the other hand patients, will both receive an active study treatment as well as non-active study treatment (placebo). Hence, using the active treatment may experience pain relief during the investigation period. It is our confidence that these advances counterbalance any risks or discomfort obtained during the study. Some patients may experience shortness of breath and hoarseness, muscle twitching during the 2-minute stimulation, tingling, and minor skin irritation. To address the theoretical risk of vagus nerve overstimulation on the heart and airways, a study was conducted at the Lovelace Respiratory Research Institute (Albuquerque, NM) in hypersensitized beagles. Review of heart rate and airway resistance before, during and after stimulation indicated that there were no significant adverse changes associated with stimulation. These results are consistent with the human clinical experience with the GammaCore device, together with ECG data from over 20 subjects as additional safety data specific to the potential cardiovascular risks of nVNS treatment (Busch et al., 2013).

Currently approximately 2000 GammaCore devices have been used in European countries to date. Physicians were asked to voluntarily provide feedback on the outcomes observed in their patients for whom the commercial GammaCore device was prescribed. Patients have safely self-administered stimulation using the GammaCore device between 4 and 20 times per day using a prophylactic and/or acute treatment protocol. In the countries in which the GammaCore is marketed, there have been 167 total complaint reports from sales / distribution (between July 2011 to August 24, 2015) identified through the company’s complaint handling system or in discussions with physicians who prescribed the devices for their patients. Seventy-two reports are classified as medical events (of those, 27 are reports of “treatment not effective”). None were device related

serious adverse events. Hence, we strongly believe, that risk of using GammaCore is very minimal, and that the treatment advances counterbalance any discomfort which may occur.

The patients will receive no economical compensation for participation in this trial. However, rules of compensation for travelling expenses apply. The descriptive nature of the study may not benefit the participating patients, as they will receive treatment according to standard procedures, as soon as they are adequately diagnosed. We believe that results, whenever positive, negative or inconclusive obtained from the study, will provide new insight to the underlying mechanisms of vagal stimulation and its influence on the pain mechanisms, and hence may provide future treatment options for chronic pancreatitis.

Patients will continue on their usual analgesic medication when the trial ends. This also applies for dropouts.

11.4 Patient information and consent

The informed consent form must be in compliance with ICH GCP, local regulatory requirements, and legal requirements. The sponsor or by delegation sub-investigators must ensure that each trial subject is fully informed about the nature and objectives of the trial and possible risks associated with participation. The investigator or by delegation sub-investigators will obtain written informed consent from each subject before any trial-specific activity is performed. The investigator will retain the original of each subject's signed consent form in up to 5 years. Data will be stored at the department of Radiology specific back-up drive, under the responsibility of Jens Brøndum Frøkjær.

A research biobank will be created during this study in order to review and report as determined by paragraph 4.1.1.c ("Sundhedsvidenskabelige forskningsprojekter"). After the end of the study (efter endt forsøg) this biological material will be transferred to a biobank in case of future research. In the case of further research the participants must give a separate consent to allow this.

11.5 Study Initiation

Before initiating the study, the investigator will have written and dated approval from the Danish Health and Medicines Authority and the Central Denmark Region Committee on Health Research Ethics for the study protocol and any amendments, written informed consent form, consent form updates, and any other written information to be provided the participants. Approval will be indicated in writing with reference to the final protocol number and date. During the study the investigator should provide all documents that are subject to review.

11.6 Study end report

A report will be submitted for the Danish Health and Medicines Authority and The North Denmark Region Committee on Health Research Ethics at the end of the trial as requested by law. The investigator will inform the Danish Health and Medicines Authority and The North Denmark Committee on Health Research Ethics of the trial. No later than 90 days after trial termination, the "Declaration of the end of trial form" has to be submitted to The Danish Health and Medicines Authority. End of the study will reported no later than 3 months after the study termination to The North Denmark Region Committee on Health Research Ethics.

11.7 Trial monitoring

During trial conduct, the Good Clinical Practice unit (GCP) will conduct periodic monitoring of all signed consents at monitoring visits to ensure that the protocol and GCP standards are followed. The monitors may review source documents and medical records to confirm that data recorded on CRFs is accurate. Thus, GCP monitoring includes all signed consents, signed power of attorney (fuldmagtserklæring), and adverse events.

11.8 Trial audits and inspections

The investigator and institution will allow the GCP unit and appropriate regulatory authorities direct access to source documents to perform this verification. The trial site may be subject to review by the institutional review board, the North Denmark Region Committee on Health Research Ethics, and/or to quality assurance audits performed by the GCP unit, and/or to inspection by appropriate regulatory authorities. It is important that the investigator(s) and their relevant personnel are available during the monitoring visits and possible audits or inspections and that sufficient time is devoted to the process.

11.10 Finances

The initiative to this study project comes from the Principal Investigator/sponsor and is independent of the company producing the medical device. All the medical devices will be bought by the principal investigator. Hence, the company producing the medical device do not have any conflict of interest. The project has received financial support from "Det Obelske Familiefond" for post.doc. salary (1350000 kr.), and "Axel Muusfeldts Fond" (100000 kr.) and "Læge Sofus Carl Emil Friis og Hustru Olga Doris Friis' Legat" (62200 kr.) for purchasing equipment such as the stimulation devices. These financial supports do not have any specific rights related to publication of the results, and positive as well as negative and inconclusive, will be published in scientific journals. The researchers have no financial interests in the project, their salary is secured with help from the University and Hospital. Every researcher (incl. doctors, Ph.D. student, senior researchers, nurses, radiographers) is employed at Aalborg University or Aalborg University Hospital. MRI scanner facilities and

experimental laboratory facilities are provided by the Radiology Research Unit and Center for pancreatic Diseases at Aalborg University Hospital. The participants in the study will not be paid for participation but there will be compensation for transport.

12. DISSEMINATION

All publication rights belong to the principal investigator and sponsor. Positive as well as negative and inconclusive trial results will be published in international peer-reviewed journals in the field of gastroenterology, neuroimaging, biochemistry, and inflammation. A primary author will be subscribed according to the Vancouver system. The Danish Health and Medicines Authority and the Central Denmark Region Committee on Health Research Ethics.

13. REFERENCES

All, N. T. B. C. (n.d.). T r anscutaneous stimulation of the cervical br branch anch of the vagus nerv nerve e for cluster headache and migr migraine aine, 1–4.

Andersen, B. N., Pedersen, N. T., Scheel, J., & Worning, H. (1982). Incidence of alcoholic chronic pancreatitis in Copenhagen. *Scandinavian Journal of Gastroenterology*, *17*(2), 247–52. https://doi.org/10.3109/00365528209182047

Andren-Sandberg, A., Ansorge, C., Eiriksson, K., Glomsaker, T., & Maleckas, A. (2005). Treatment of pancreatic pseudocysts. *Scandinavian Journal of Surgery : SJS : Official Organ for the Finnish Surgical Society and the Scandinavian Surgical Society*, *94*(2), 165–175.

Bigger, J. T., Fleiss, J. L., Rolnitzky, L. M., & Steinman, R. C. (1993). The ability of several short-term measures of RR variability to predict mortality after myocardial infarction. *Circulation*, *88*(3), 927–934. https://doi.org/10.1161/01.CIR.88.3.927

Busch, V., Zeman, F., Heckel, A., Menne, F., Ellrich, J., & Eichhammer, P. (2013). The effect of transcutaneous vagus nerve stimulation on pain perception-An experimental study. *Brain Stimulation*, *6*(2), 202–209. https://doi.org/10.1016/j.brs.2012.04.006

Chakravarthy, K., Chaudhry, H., Williams, K., & Christo, P. J. (2015). Review of the Uses of Vagal Nerve Stimulation in Chronic Pain Management. *Current Pain and Headache Reports*. https://doi.org/10.1007/s11916-015-0528-6

Coviello, I., Pinnacchio, G., Laurito, M., Stazi, A., Battipaglia, I., Barone, L., … Crea, F. (2013). Prognostic role of heart rate variability in patients with ST-segment elevation acute myocardial infarction treated by primary angioplasty. *Cardiology*, *124*(1), 63–70. https://doi.org/10.1159/000345779

Drewes, A. M., Krarup, A. L., Detlefsen, S., Malmstrøm, M.-L., Dimcevski, G., & Funch-Jensen, P. (2008). Pain in chronic pancreatitis: the role of neuropathic pain mechanisms. *Gut*, *57*(11), 1616–1627. https://doi.org/10.1136/gut.2007.146621

ElectroCore. (2017a). About us. Retrieved from http://www.electrocore.com/about-us

ElectroCore. (2017b). Active Therapy Areas. Retrieved from http://www.electrocore.com/active-therapy-areas

Evrengul, H., Dursunoglu, D., Cobankara, V., Polat, B., Seleci, D., Kabukcu, S., … Kilic, M. (2004). Heart rate variability in patients with rheumatoid arthritis. *Rheumatology International*, *24*(4), 198–202. https://doi.org/10.1007/s00296-003-0357-5

Farmer, A. D., Coen, S. J., Kano, M., Weltens, N., Ly, H. G., Botha, C., … Aziz, Q. (2014). Normal values and reproducibility of the real-time index of vagal tone in healthy humans: a multi-center study. *Annals of Gastroenterology : Quarterly Publication of the Hellenic Society of Gastroenterology*, *27*(4), 362–368.

Frangos, E., Ellrich, J., & Komisaruk, B. R. (2015). Non-invasive access to the vagus nerve central projections via electrical stimulation of the external ear: FMRI evidence in humans. *Brain Stimulation*, *8*(3), 624–636. https://doi.org/10.1016/j.brs.2014.11.018

Frokjaer, J. B., Brock, C., Softeland, E., Dimcevski, G., Gregersen, H., Simren, M., & M Drewes, A. (2013). Macrostructural brain changes in patients with longstanding type 1 diabetes mellitus - a cortical thickness analysis study. *Experimental and Clinical Endocrinology & Diabetes : Official Journal, German Society of Endocrinology [and] German Diabetes Association*, *121*(6), 354–360. https://doi.org/10.1055/s-0033-1345120

Frokjaer, J. B., Olesen, S. S., Gram, M., Yavarian, Y., Bouwense, S. a. W., Wilder-Smith, O. H. G., &

Drewes, a. M. (2011). Altered brain microstructure assessed by diffusion tensor imaging in patients with chronic pancreatitis. *Gut*, *60*(11), 1554–1562. https://doi.org/10.1136/gut.2010.236620

Frøkjær, J. B., Bouwense, S. A. W., Olesen, S. S., Lundager, F. H., Eskildsen, S. F., van Goor, H., … Drewes, A. M. (2012). Reduced Cortical Thickness of Brain Areas Involved in Pain Processing in Patients With Chronic Pancreatitis. *Clinical Gastroenterology and Hepatology*, *10*(4), 434–438. https://doi.org/10.1016/j.cgh.2011.11.024

Gaul, C., Diener, H.-C., Silver, N., Magis, D., Reuter, U., Andersson, A., … Straube, A. (2016). Non-invasive vagus nerve stimulation for PREVention and Acute treatment of chronic cluster headache (PREVA): A randomised controlled study. *Cephalalgia : An International Journal of Headache*, *36*(6), 534–546. https://doi.org/10.1177/0333102415607070

Grover, M., & Drossman, D. A. (2010). Functional abdominal pain. *Current Gastroenterology Reports*, *12*(5), 391–398. https://doi.org/10.1007/s11894-010-0125-0

Groves, D. A., & Brown, V. J. (2005). Vagal nerve stimulation: A review of its applications and potential mechanisms that mediate its clinical effects. *Neuroscience and Biobehavioral Reviews*, *29*(3), 493–500. https://doi.org/10.1016/j.neubiorev.2005.01.004

Handforth, A., DeGiorgio, C. M., Schachter, S. C., Uthman, B. M., Naritoku, D. K., Tecoma, E. S., … Wheless, J. W. (1998). Vagus nerve stimulation therapy for partial-onset seizures: a randomized active-control trial. *Neurology*, *51*(1), 48–55.

Hansen, T. M., Olesen, A. E., Simonsen, C. W., Drewes, A. M., & Frokjaer, J. B. (2014). Cingulate metabolites during pain and morphine treatment as assessed by magnetic resonance spectroscopy. *Journal of Pain Research*, *7*, 269–276. https://doi.org/10.2147/JPR.S61193

Kinfe, T. M., Pintea, B., Muhammad, S., Zaremba, S., Roeske, S., Simon, B. J., & Vatter, H. (2015). Cervical non-invasive vagus nerve stimulation (nVNS) for preventive and acute treatment of episodic and chronic migraine and migraine-associated sleep disturbance: a prospective observational cohort study. *The Journal of Headache and Pain*, *16*, 101. https://doi.org/10.1186/s10194-015-0582-9

Kraus, T., Kiess, O., H??sl, K., Terekhin, P., Kornhuber, J., & Forster, C. (2013). CNS BOLD fMRI effects of sham-controlled transcutaneous electrical nerve stimulation in the left outer auditory canal - A pilot study. *Brain Stimulation*, *6*(5), 798–804. https://doi.org/10.1016/j.brs.2013.01.011

Lange, G., Janal, M. N., Maniker, A., Fitzgibbons, J., Fobler, M., Cook, D., & Natelson, B. H. (2011). Safety and efficacy of vagus nerve stimulation in fibromyalgia: a phase I/II proof of concept trial. *Pain Medicine (Malden, Mass.)*, *12*(9), 1406–1413. https://doi.org/10.1111/j.1526-4637.2011.01203.x

Lanza, G. A., Guido, V., Galeazzi, M. M., Mustilli, M., Natali, R., Ierardi, C., … Maseri, A. (1998). Prognostic role of heart rate variability in patients with a recent acute myocardial infarction. *The American Journal of Cardiology*, *82*(11), 1323–1328.

Laversuch, C. J., Seo, H., Modarres, H., Collins, D. A., McKenna, W., & Bourke, B. E. (1997). Reduction in heart rate variability in patients with systemic lupus erythematosus. *The Journal of Rheumatology*, *24*(8), 1540–1544.

Lieb 2nd, J. G., Forsmark, C. E., Lieb, J. G., & Forsmark, C. E. (2009). Review article: pain and chronic pancreatitis. *Aliment Pharmacol Ther*, *29*(7), 706–19. https://doi.org/10.1111/j.1365-2036.2009.03931.x

Lindgren, S., Stewenius, J., Sjolund, K., Lilja, B., & Sundkvist, G. (1993). Autonomic vagal nerve dysfunction in patients with ulcerative colitis. *Scandinavian Journal of Gastroenterology*, *28*(7), 638–642.

Makikallio, T. H., Huikuri, H. V, Makikallio, A., Sourander, L. B., Mitrani, R. D., Castellanos, A., &

Myerburg, R. J. (2001). Prediction of sudden cardiac death by fractal analysis of heart rate variability in elderly subjects. *Journal of the American College of Cardiology*, *37*(5), 1395–1402.

Napadow, V., Edwards, R. R., Cahalan, C. M., Mensing, G., Greenbaum, S., Valovska, A., … Wasan, A. D. (2012). Evoked Pain Analgesia in Chronic Pelvic Pain Patients Using Respiratory-Gated Auricular Vagal Afferent Nerve Stimulation. *Pain Medicine (United States)*, *13*(6), 777–789. https://doi.org/10.1111/j.1526-4637.2012.01385.x

NICE. (2016). T r anscutaneous stimulation of the cervical br branch anch of the vagus nerv nerve e for cluster headache and migr migraine aine, 2–9. Retrieved from https://www.nice.org.uk/guidance/IPG552/chapter/1-Recommendations

Nierenberg, A. A., Alpert, J. E., Gardner-Schuster, E. E., Seay, S., & Mischoulon, D. (2008). Vagus nerve stimulation: 2-year outcomes for bipolar versus unipolar treatment-resistant depression. *Biological Psychiatry*, *64*(6), 455–460. https://doi.org/10.1016/j.biopsych.2008.04.036

Olesen, S. S., Frøkjær, J. B., Lelic, D., Valeriani, M., & Drewes, A. M. (2011). Pain-associated adaptive cortical reorganisation in chronic pancreatitis. *Pancreatology*, *10*(6), 742–751. https://doi.org/10.1159/000321644

Olesen, S. S., Graversen, C., Olesen, A. E., Frøkjær, J. B., Wilder-Smith, O., Van Goor, H., … Drewes, A. M. (2011). Randomised clinical trial: Pregabalin attenuates experimental visceral pain through sub-cortical mechanisms in patients with painful chronic pancreatitis. *Alimentary Pharmacology and Therapeutics*, *34*(8), 878–887. https://doi.org/10.1111/j.1365-2036.2011.04802.x

Oshinsky, M. L., Murphy, A. L., Hekierski, H. J., Cooper, M., & Simon, B. J. (2014). Noninvasive vagus nerve stimulation as treatment for trigeminal allodynia. *Pain*, *155*(5), 1037–1042. https://doi.org/10.1016/j.pain.2014.02.009

Pasricha, P. J. (2012). Unraveling the mystery of pain in chronic pancreatitis. *Nature Reviews. Gastroenterology & Hepatology*, *9*(3), 140–51. https://doi.org/10.1038/nrgastro.2011.274

Poulsen, J. L., Olesen, S. S., Malver, L. P., Frokjaer, J. B., & Drewes, A. M. (2013). Pain and chronic pancreatitis: a complex interplay of multiple mechanisms. *World Journal of Gastroenterology : WJG*, *19*(42), 7282–7291. https://doi.org/10.3748/wjg.v19.i42.7282

Ring, H. A., White, S., Costa, D. C., Pottinger, R., Dick, J. P. R., Koeze, T., & Sutcliffe, J. (2000). A SPECT study of the effect of vagal nerve stimulation on thalamic activity in patients with epilepsy. *Seizure*, *9*(6), 380–384. https://doi.org/10.1053/seiz.2000.0438

Silberstein, S. D., Calhoun, A. H., Lipton, R. B., Grosberg, B. M., Cady, R. K., Dorlas, S., … Saper, J. R. (2016). Chronic migraine headache prevention with noninvasive vagus nerve stimulation: The EVENT study. *Neurology*, *87*(5), 529–538. https://doi.org/10.1212/WNL.0000000000002918

Silberstein, S. D., Mechtler, L. L., Kudrow, D. B., Calhoun, A. H., McClure, C., Saper, J. R., … Tepper, S. J. (2016). Non???Invasive Vagus Nerve Stimulation for the ACute Treatment of Cluster Headache: Findings From the Randomized, Double-Blind, Sham-Controlled ACT1 Study. *Headache*, *56*(8), 1317–1332. https://doi.org/10.1111/head.12896

Steer, M. L., Waxman, I., & Freedman, S. (1995). Chronic pancreatitis. *New England Journal of Medicine*.

Stein, K. S., McFarlane, I. C., Goldberg, N., & Ginzler, E. M. (1996). Heart rate variability in patients with systemic lupus erythematosus. *Lupus*, *5*(1), 44–48.

Thayer, J. F. (2009). Vagal tone and the inflammatory reflex. *Cleveland Clinic Journal of Medicine*, *76 Suppl 2*, S23-6. https://doi.org/10.3949/ccjm.76.s2.05

van Esch, A. A., Wilder-Smith, O. H., Jansen, J. B., van Goor, H., & Drenth, J. P. (2006). Pharmacological

management of pain in chronic pancreatitis. *Dig Liver Dis*, *38*(7), 518–526. https://doi.org/S1590-8658(06)00056-9 [pii]\r10.1016/j.dld.2006.02.002 [doi]

Woolf, C. J., & Mannion, R. J. (1999). Neuropathic pain: Aetiology, symptoms, mechanisms, and management. *Lancet*. https://doi.org/10.1016/S0140-6736(99)01307-0

Yuan, H., & Silberstein, S. D. (2017). Vagus Nerve Stimulation and Headache. *Headache*, *57 Suppl 1*, 29–33. https://doi.org/10.1111/head.12721

14. APPENDIX

Figure 1: Study design

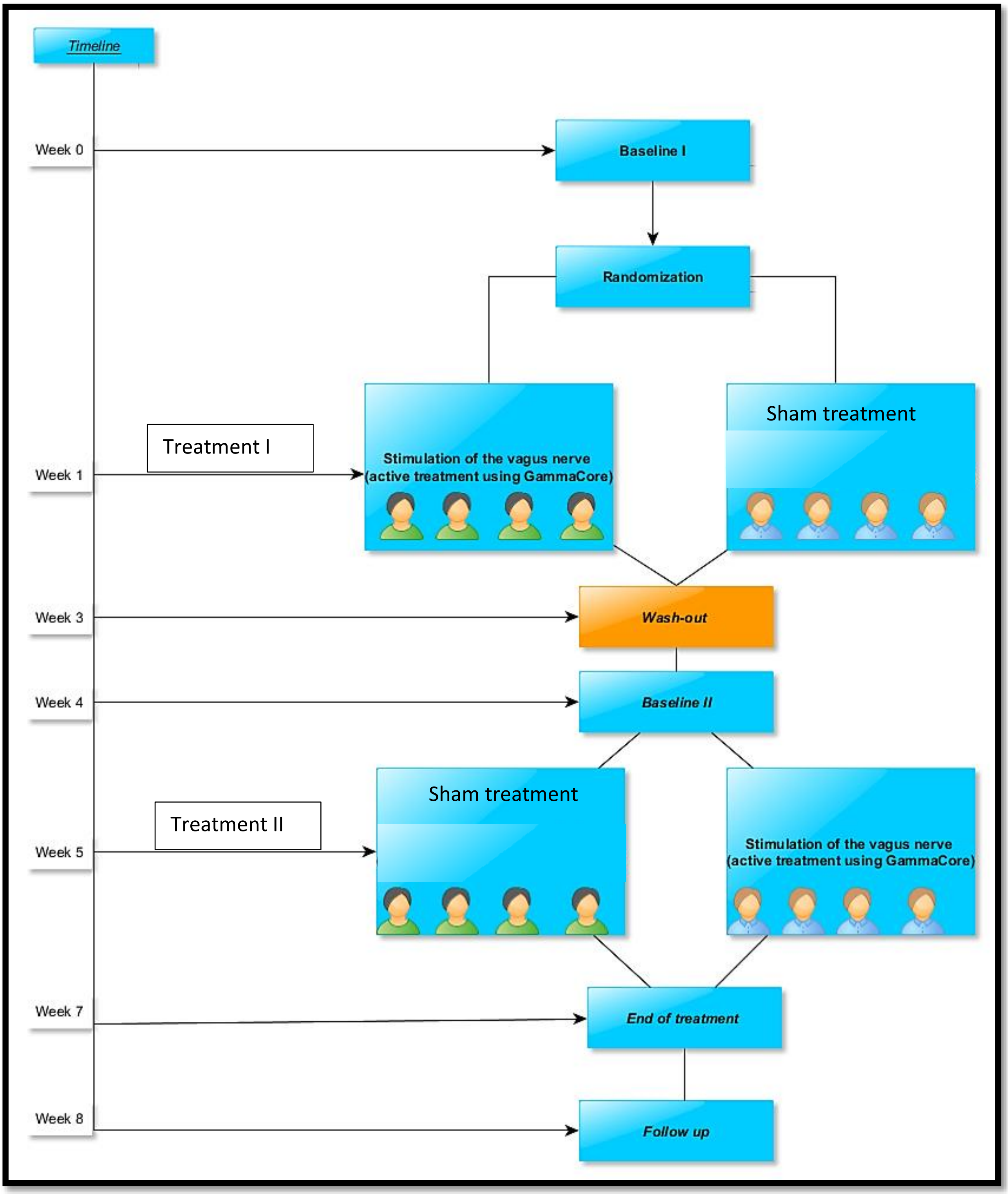

Supplement: S1 Appendix — (PDF) [file pone.0247653.s004.pdf]
