# Supplementary material for: Cervical transcutaneous vagal neuromodulation in chronic pancreatitis patients with chronic pain: A randomised sham controlled clinical trial
Source: PLoS One. 2021 Feb 26;16(2):e0247653. doi: 10.1371/journal.pone.0247653 (PMC7909707; doi:10.1371/journal.pone.0247653)
Supplement: Attachment — Submitted filename: Response to reviewers PONE_D-20-13163-R1 030221.docx [file pone.0247653.s006.docx]

**Response to comments:**

The editor wrote #1: “The revisions that need to be made relate to your smaller sample size.

You state that your team found no difference between the groups in this study, however as your sample size is small, it is possible that your test is underpowered. You do state this limitation briefly in your manuscript. However it would be more correct to state throughout your manuscript that you found no evidence of a difference between groups (as opposed to finding evidence of no difference between groups). As such, please re-word some of your statements made in your manuscript as follows:

Lines 68-69: Current statement: "In this sham-controlled crossover study, two weeks transcutaneous vagal nerve stimulation did not induce pain relief in patients with chronic pancreatitis."

Suggested statement: "In this sham-controlled crossover study, we found no evidence that two weeks transcutaneous vagal nerve stimulation induces pain relief in patients with chronic pancreatitis."

Lines 388-390: Current statement: "Our sham-controlled crossover study provided evidence that adjuvant treatment with two weeks nVNS did not induce pain relief as compared to sham treatment in patients with painful chronic pancreatitis"

Suggested statement: "Our sham-controlled crossover study provided no evidence that adjuvant treatment with two weeks nVNS induces pain relief as compared to sham treatment in patients with painful chronic pancreatitis"”

Our reply: Thanks for this helpful comment. We agree, due to the smaller sample size, that it is more correct to conclude that we found no evidence of difference between groups/treatments. We now reviewed the entire manuscript to adopt this. We agree that this concerns the two concluding statements (lines 68-69 and 388-390), which have now been corrected as suggested. In the beginning of the Conclusion section, we also adjusted lines 280-281 now reading “Overall, we found no evidence of significant difference in pain relief between the two treatments” and lines 288-290 reading “In contrast to our hypothesis, we found no evidence that CP patients had a significant pain reduction comparing the nVNS and sham treatments”.

The editor wrote #2: “In addition, please add one to two sentences about your sample size limitation to the limitations section of your discussion.”

Our reply: We now added the sample size discussion in the beginning of the limitations section, now reading: “First, even though a sample size calculation was performed prior to this study, such calculations in clinical studies with experimental interventions are often based on some uncertain assumptions. Hence, we could have a too small sample with a risk of false negative results regarding our primary outcomes”.

The editor wrote #3: “Finally, we request that you revise the phrase "the effects of" in your manuscript title.

Our reply: We agree. The title is now rephrased avoiding the “effect of” statement. It now simply reads: “Cervical transcutaneous vagal neuromodulation in chronic pancreatitis patients with chronic pain: a randomised sham controlled clinical trial”.
